# Development, Reliability, and Initial Validation of the Fear of Institutional Betrayal Questionnaire

**DOI:** 10.1177/03616843261449772

**Published:** 2026-05-13

**Authors:** Gena K. Dufour

**Affiliations:** 1 Department of Psychology, 8637 the University of Windsor, Windsor, Canada

**Keywords:** institutional betrayal, campus sexual violence, scale development, factor analysis

## Abstract

*Fear of institutional betrayal* is a new conceptual extension of the original institutional betrayal framework. The original framework is a way of making sense of, and giving credibility to, the experiences of harm perpetrated by institutions. The original authors developed a checklist of institutional actions or inactions that harm survivors of sexual violence. Building on this work, fear of institutional betrayal is an attitudinal construct that represents the belief that post-secondary institutions will inevitably fail to prevent or acceptably respond to campus sexual violence, and that engagement with the institution in the aftermath of campus sexual violence will result in harm for survivors. In this paper, two studies provided evidence for the psychometric soundness of the Fear of Institutional Betrayal Questionnaire (FIBQ). The first study established the scale's reliability and unidimensional factor structure. A replication study in a larger, more diverse, national sample of students confirmed these findings. Construct validity was supported across both studies by theoretically consistent correlations and expected group differences across demographic characteristics. These studies demonstrate that the FIBQ is a psychometrically sound instrument that captures a construct not previously assessed in the literature.

When sexual violence happens on campus, the ways in which an institution responds to that incident can sometimes perpetuate additional harm for survivors and others in the university community, a phenomenon known as i*nstitutional betrayal* (Smith & Freyd, 2013, 2014, 2017). Examples of institutional betrayal are commonly shared in the media. For instance, in 2015, a student athlete named Brock Turner sexually assaulted a woman named Chanel Miller outside of a fraternity party at Stanford University (Mascagni, 2017; Miller, 2020). This event caught the attention of the media in North America and globally, highlighting several troubling problems with the investigation and response procedures used by post-secondary institutions. The dehumanizing, silencing, and traumatizing investigation and legal processes experienced by Chanel were outlined in her memoir (Miller, 2020). Examples like this and others that have attracted widespread media attention (e.g., Campbell et al., 2023; Smith & Freyd, 2014) illustrate glaring problems with post-secondary institutions’ responses to incidents of violence.

As stories like Chanel Miller's are publicized, academic scholarship has begun to explore public perceptions of institutional responses to violence (e.g., Christl et al., 2024; Mancini et al., 2019; PettyJohn et al., 2023). One trend has become increasingly noticeable in this area: many students and other stakeholders in post-secondary settings worry that their institutions would not be supportive in the event of an assault or other act of violence (Kettrey et al., 2026; Stewart, 2021). Moreover, students are also increasingly aware and wary of the structural and cultural conditions present in institutional contexts that contribute to sexual violence (Gretgrix & Farmer, 2026; PettyJohn et al., 2023; Riley, 2019; Rizzo et al., 2021). In particular, these fears are particularly salient for students from minority groups, such as graduate students, international students, racialized students, or other minorities (e.g., Bloom et al., 2023; Karunaratne, 2021; Kettrey et al., 2026).

## Conceptualizing Fear of Institutional Betrayal

The current study introduces a new application of the institutional betrayal framework, a concept called *fear of institutional betrayal.* Although the original institutional betrayal framework describes how the actions and inactions of a university (or other institutions) can perpetuate further harm to survivors of sexual violence in the wake of trauma (Smith & Freyd, 2013, 2014, 2017, for a review, also see Christl et al., 2024), fear of institutional betrayal is an attitudinal concept that describes how individuals might fear that these actions would happen in the future. Specifically, fear of institutional betrayal is the belief held by members of an institution that the institution will *inevitably* fail to prevent or acceptably respond to an instance of campus sexual violence, and that engagement with the institution in the aftermath of sexual violence will result in further harm for survivors. In essence, fear of institutional betrayal is the fear that involvement with the institution after an incident of violence will make things worse for the survivor. Fear of institutional betrayal also reflects concerns about the structural and cultural conditions that precede assault or disclosure. In the context of campus sexual violence, “members of an institution” are most commonly students, but may also reflect the experiences of faculty, staff, and other university community members.

Fear of institutional betrayal is an implicitly held attitude. People may believe their institutions’ responses to the issue of sexual violence may be insufficient, even if they do not label this belief as a “fear.” However, participants in various studies have explicitly reported that they are *afraid* of the unknown consequences of disclosing to their institution that they have been victimized (Cantor et al., 2020; Dufour, 2024; Karunaratne, 2021; PettyJohn et al., 2023). In the institutional betrayal literature, several scholars have directly or indirectly called attention to individuals in post-secondary environments being *afraid* of institutional betrayal (e.g., Dufour, 2024; Reffi et al., 2021; Rosenthal et al., 2017; Spencer et al., 2017; Walsh, 2026). Accordingly, institutional betrayal is an appropriate foundational framework for exploring this phenomenon, and the use of the word “*fear*” in this context is appropriate for capturing the experiences of survivors and others who hold these perceptions.

It is possible that fear of institutional betrayal may influence help-seeking, disclosure, and perceptions of institutional legitimacy (Gray, 2024; Walsh, 2026). Moreover, because fear of institutional betrayal includes concerns about poor institutional preparation and the prevention of sexual violence, it can be understood as a climate-level concern (Cantor et al., 2020; Kettrey et al., 2026; Swartout et al., 2019), with implications for feminist understandings of institutional legitimacy, power, and accountability (Gretgrix & Farmer, 2026). The goal of this research was to develop a questionnaire that would provide researchers with a standardized way of assessing these beliefs. This new tool, the Fear of Institutional Betrayal Questionnaire (FIBQ), has applications for understanding campus climate around sexual violence.

## What is There to be Afraid of?

To develop the FIBQ, it was necessary to review the specific actions, inactions, or outcomes identified in previous literature as sources of fear. This inquiry informed the development of the scale by isolating recurring trends in campus sexual violence response literature regarding the anxieties and fears about expected responses, which were then used as items in the questionnaire. The extent and nature of expected harm varies by individual, but largely encompasses a fear of unsupportive or insensitive institutional responses to specific instances of violence, or fears of general lack of institutional preparedness about sexual violence in general (Amar et al., 2014; Brink et al., 2021; Cantor et al., 2020).

Fears of institutional betrayal apply to all stages of the reporting and investigative process. For many, anxieties begin with sexual assault disclosure. Students commonly note that they fear their disclosures will be minimized, dismissed, or not taken seriously (Cantor et al., 2020; Rosenthal et al., 2017; Sall & Littleton, 2022; Tredinnick, 2022), and many students fear they will not be believed at all (Boyle et al., 2017; Sall & Littleton, 2022; Spencer et al., 2017). Fear of victim-blaming responses is another widely reported concern (Stader & Williams-Cunningham, 2017). Conversely, other survivors express that, even if they are believed, nothing will be done to address the assault, and so reporting is perhaps more trouble than it is worth (Brink et al., 2021; Hibberd, 2017; Nightingale, 2021). Survivors have articulated concerns both about the ability of individual institutional representatives to respond effectively (Nightingale, 2021) and about systemic limitations that impede institutional-level reform (Brink et al., 2021).

In the event of an investigation, students commonly report that they do not trust the university to conduct a fair investigation (Tredinnick, 2022), and a fear of bias within those investigations is particularly salient for some individuals from marginalized groups (Tredinnick, 2022). Some believe that cases will be mishandled in unpredictable ways (Rosenthal et al., 2017), and thus, a fear of unknown blowback or consequences during an investigation outweighs any perceived benefits. Fear of reprisal is also a major concern, including for would-be whistleblowers who report being afraid to come forward (Ahern, 2018; Sall & Littleton, 2022; Smith & Freyd, 2014; Webermann & Holland, 2022).

Other times, fears about the institution may be related to other aspects of a survivor's (intersectional) identity (e.g., also a person of color) or are specific to the circumstances of the survivor. For example, students whose first language is not English may have concerns regarding the institution's ability to help them in their first language, or provide culturally appropriate services (Hibberd, 2017). Fear of judgment or prejudice is of particular concern to some survivors, such as if an LGBT student reports a same-sex assault (Hackman et al., 2022). Some research has also found that students from certain groups fear losing their social support circles (Linder et al., 2019). Survivors may fear losing LGBT centers, women's centers, or other minority student spaces (Karunaratne, 2021; Nightingale, 2022). Identity-focused fears may be especially prevalent on campuses characterized by pervasive racism or other forms of prejudice (Kettrey et al., 2026).

Often, students disclose fears around confidentiality (Amar et al., 2014; Cantor et al., 2020), specifically noting concerns that if they share information about an assault to a formal support, the information may not be anonymous or confidential, or that campus service providers might share personal information about them without consent (Hibberd, 2017; Javorka & Campbell, 2019). Concerns around confidentiality are often directly linked to fears regarding the perpetrator: For many survivors, it is a direct concern that, should any information make its way back to the perpetrator, that individual may act out, seek revenge, or otherwise harass the survivor in some capacity (Amar et al., 2014; Spencer et al., 2017).

Fear of institutional reprisal (Boyle et al., 2017) is also commonly discussed in the literature. For instance, sexual violence often happens in the context of undergraduate parties. Survivors often fear reprisal or punishment for having engaged in partying, drinking, using illegal substances, or other similar behaviors in campus settings (Stader & Williams-Cunningham, 2017). Other times, students fear that reporting an assault may have other unforeseen academic consequences (Spencer et al., 2017), such as being forced to drop out of classes or delay graduation. Fear of institutional reprisal is also of concern to potential whistleblowers within an institution (Ahern, 2018). Fear of institutional betrayal can therefore also manifest in individuals who are not directly involved in campus sexual violence incidents but may be in a position to intervene or report instances that have occurred (Webermann & Murphy, 2022), or otherwise call attention to poor institutional responses to violence (Ahern, 2018).

Some survivors express a fear that reporting to the institution inherently means the police will become involved (Javorka & Campbell, 2021). These fears are sometimes rooted in historical fears of police mistreatment and disbelief, particularly for survivors with marginalized identities (Hackman et al., 2022; Javorka & Campbell, 2021; Miller, 2020). Many survivors are generally unwilling to put themselves at risk of secondary victimization at the hands of law enforcement, who may become involved in their case (Hackman et al., 2022; Miller, 2020).

Finally, students’ fears may also encompass broader anxieties about whether the institution is adequately prepared to prevent sexual violence in the first place (Amar et al., 2014; Christl et al., 2024). There is extensive scholarship that describes the insufficient, underfunded, or poorly structured systems in place on post-secondary campuses that require structural reform and investment in order to comprehensively address gender-based violence (Amar et al., 2014; Gretgrix & Farmer, 2026; Phipps, 2020; Riley, 2019; Tredinnick, 2022; White House Task Force to Protect Students from Sexual Assault, 2014). Research on bystander theory, bystander intervention, and sexual assault prevention is rooted in the acknowledgement that institutions need infrastructure and tangible commitment in order to meaningfully address sexual violence (Gretgrix & Farmer, 2026; Rizzo et al., 2021; Tredinnick, 2022). The emerging body of research on institutional courage (Center for Institutional Courage, 2024; Gómez et al., 2023; Smidt et al., 2023) also demonstrates cultural awareness of institutional climate, preparedness, and structural-level considerations in this capacity. These documented structural limitations provide the conditions that may drive fear of institutional betrayal at a cultural level.

## Institutional Betrayal and the Institutional Betrayal Questionnaire

The original institutional betrayal framework (see Smith & Freyd, 2014) conceptualized institutional behavior as occurring on multiple dimensions. Specifically, the authors explained that institutional betrayal includes both acts of omission (e.g., failure to take steps to systematically prevent sexual violence on campus) as well as acts of commission (e.g., creating policies that make it harder for survivors to formally report incidents of violence). The authors also posited that although some elements of institutional betrayal might be “apparently isolated” events (e.g., a poor interpersonal reaction), some forms are “apparently systemic” (e.g., a lack of policies about prevention).

There are several different tools designed to measure the experiences of institutional betrayal directly. Using the dimensions described, Jennifer Freyd and colleagues (Smith & Freyd, 2013, 2014, 2017) originally developed the Institutional Betrayal Questionnaire (IBQ) to provide researchers with a way to quantify and measure institutional betrayal. Participants in the original study (Smith & Freyd, 2013) were provided with a list of institutional actions and inactions, such as “Not taking proactive steps to prevent this type of experience,” to create a list of betrayal experiences. The authors noted that, similar to other checklists of traumatic life experiences, the IBQ is more like a census or checklist rather than a scale meant to measure an underlying attitude. However, the IBQ laid the groundwork for several derivative scales (see Center for Institutional Courage, 2024). The original 7-item questionnaire has been updated and modified in various ways, including the IBQ.2 (which added five new items and changed some wordings; Smith & Freyd, 2017) and the Institutional Betrayal and Support Questionnaire (IBSQ), which added additional questions about supportive responses as well (Center for Institutional Courage, 2024; Rosenthal et al., 2016).

Since the development of the first IBQ, the ways in which researchers have used this tool vary significantly, and nearly every study that has used the IBQ has modified it in some way (Lind et al., 2020; Rosenthal et al., 2016; Rosenthal et al., 2017; Smidt et al., 2021; Smidt et al., 2023). Several authors have also used the institutional betrayal framework as a starting place for developing theoretically related constructs. The most notable example of this is work by Jennifer Gómez and colleagues, which has explored the construct of cultural betrayal trauma (see Gómez, 2016; 2019; 2023; for more on this). Other research has adapted the framework to explore IB in other contexts. Tamaian and Klest (2018) developed the Institutional Betrayal Questionnaire-Medical System (IBQ-MS), which is a 42-item tool for assessing provider and institutional responses to patients’ negative healthcare experiences in medical settings (e.g., minimizing symptoms). The IBQ-MS was created by modifying the wording of the original IBQ to be specific to the medical context in Canada, and adding other items based on results from previous qualitative work (Tamaian et al., 2017).

In another example, Lind et al. (2020) developed the IBQ.Climate, which was a tool designed to assess perceptions of sexual harassment climate in high school settings. This 12-item tool is a modified version of the IBQ.2, which changed the wording of the questions to explore perceptions of climate rather than to produce a checklist of experiences that have directly happened to the respondent (e.g., the institution creates an environment in which sexual harassment seems common or normal). Items are answered on a 4-point scale and were developed specifically for the high school context. The IBQ.Climate is notable because, unlike the IBQ, IBQ.2, or IBQ-MS, the IBQ.Climate is an attitudinal scale rather than a checklist. In their study, Lind et al. (2020) reported a Cronbach's alpha of .94. However, there is currently no published research on the psychometric properties, reliability, or validity of the IBQ.Climate.

Since the development of the original scale, two studies have been published exploring the psychometric properties of the IBQ.2. Reffi et al. (2021) collected a sample of 426 mixed-gender participants from Amazon Mechanical Turk (MTurk). Authors reported that the IBQ.2 demonstrated acceptable convergent and discriminant validity. The authors also explored the dimensionality of the scale. In their original evaluation of the IBQ, Smith and Freyd (2013) found no evidence of multi-dimensionality. They suggested the original IBQ, therefore, reflected only a single, holistic factor structure. However, Reffi and colleagues’ (2021) found factor-analytic evidence of a two-factor structure: some of the questions reflected an institution's failure to prevent sexual violence, while other questions reflected an institution's response to instances of violence that have already occurred, factors they labeled as “leading to sexual victimization” and “response to sexual victimization,” respectively (p. 5669). This two-factor structure mirrored results reported by authors of the IBQ-MS (Tamaian & Klest, 2018).

Another psychometric evaluation study of the IBQ.2 was published by Monteith et al. (2021), which used Rasch Analysis to better examine the factor structure of the IBQ.2. They used a sample of 235 military sexual trauma survivors. This study found initial exploratory evidence for a three-factor model: (a) environmental factors that contribute to the likelihood of sexual violence occurring, (b) institutional responses to instances of sexual violence, and (c) perceptions of institutional belongingness following sexual violence. Thus, given that psychometric research on the IBQ has yet to consistently establish its factor structure, further investigation into these issues is necessary in emerging research.

## Theoretical and Conceptual Significance of Fear of Institutional Betrayal

Fear of institutional betrayal raises several important considerations for all members of a university community. For survivors, fear of institutional betrayal may act as a psychological barrier to disclosure and help-seeking. Webermann and Holland (2022) noted that, for survivors, institutional betrayal is not only an outcome of interacting with institutions, but can also deter engagement with institutional processes altogether. Indeed, in the extensive research on barriers to help-seeking, many studies have reported that “fear of poor institutional response” in one form or another is one of the reasons why survivors seldom report to their universities (e.g., Bloom et al., 2023; Walsh et al., 2025). In theory, fear of institutional betrayal may directly impact disclosure or service use intentions and perceptions of institutional safety, which have critical consequences for survivor wellbeing and recovery. Accordingly, fear of institutional betrayal provides quantitative insight into a potentially campus-wide fear that could lead to the structural silencing of survivors (Gray, 2024). Fear of institutional betrayal is also a community-level consideration. Fear of institutional betrayal may, for example, result from interactions with peers or institutional representatives (Riley, 2019). Mushonga et al. (2021) reported that students who heard stories about other students having negative experiences with campus reporting processes were less likely to formally report sexual violence experiences themselves. This trend reflects social norms around the gendered nature of risk (and the expectation of harm), and how women understand institutional power and harm (Lewis et al., 2018; Riley, 2019).

Notably, fear of institutional betrayal is not an attitude exclusive to survivors of violence. Any member of an institution may harbor concerns about that institution's overall capacity address campus sexual violence as a broader issue (Phipps, 2020). Consequently, fear of institutional betrayal has implications for campus climate, including community discourse about the institution's quality, and the extent to which the community believes institutional responses to campus sexual violence are adequate (Phipps, 2020; Walsh et al., 2025). Poor climate is associated with lower levels of institutional trust and a stronger sense that the institution is of low quality (Brink et al., 2021). Fear of institutional betrayal may therefore contribute to broader negative perceptions of the institution and perpetuate harmful norms around sexual violence denial, silencing, power, and patriarchy (Gray, 2024; Howard Valdivia et al., 2023).

Finally, although many institutions have implemented substantive reforms to address sexual violence in recent years (e.g., Campbell et al., 2023), positive institutional changes do not always translate into perceived legitimacy or trust among students or faculty (Gardiner & Finn, 2022; Mancini et al., 2019; Phipps, 2020; Riley, 2019). Accordingly, fear of poor response may persist even when formal policies improve. Given the potential implications of fear of institutional betrayal, the capacity to measure and track this phenomenon might enable institutions to detect climate-level mistrust and identify specific points of concern harbored by community members (e.g., fear that the perpetrator will face no consequences, or fear of a poor response based on identity).

## Development of the Fear of Institutional Betrayal Questionnaire

The items in the FIBQ were developed based on the framework of the original IBQ (Smith & Freyd, 2013), several derivative scales (e.g., Center for Institutional Courage, 2024; Lind et al., 2020; Rosenthal et al., 2017; Smith & Freyd, 2017), and additional literature in the area of perceptions of institutional responses to campus sexual violence (See supplemental materials for full list). Items from the IBQ (Smith & Freyd, 2013), the IBQ.2 (Smith & Freyd, 2017), and IBQ.Climate (Lind et al., 2020) provided the initial items that were modified for the FIBQ. To convert the items from a checklist of events to an attitudinal scale, the original items were reworded to “I am afraid that…” or “I worry that…” (e.g., “I worry that my university would make it difficult or complicated to report sexual violence”). Overall, items were constructed to either explicitly reference fear of something happening or judgments of institutional characteristics that might lead to betrayal or harm (e.g., failure to prevent violence).

The items on the FIBQ were also informed by other literature on institutional perceptions, climate, and trust, and research about reported fears from students and survivors (e.g., fear of being blamed, fear that the institution does not care about sexual violence). Similar to the FIBQ items, many of these additional items were directly modified from other validated scales assessing institutional response, trust, or climate (i.e., not IBQ scales specifically, but related measures of institutional perception). The author identified recurring concerns in the literature regarding anticipated institutional response, used the IBQ content as a baseline, and filled gaps with content modified from validated or non-validated scales in this area.

Thus, similar to Tamaian and colleagues (2017), items were added based on other relevant literature in the area of student perceptions of institutional responses to sexual violence (see supplemental materials). For instance, despite being commonly discussed across literature, there are no items on any of the IBQ scales that capture the fear that survivors will not be treated with dignity or respect by the institution (Amar et al., 2014; Brink et al., 2021; Holland, 2020; Webermann & Holland, 2022). Another example is that survivors widely report fears they will not be believed by administrators (Boyle et al., 2017; Spencer et al., 2017), but this was not previously captured by the IBQ. Thus, items were created by adapting wording from other validated scales to match the items in the pool. If no other scales contained language for an item (e.g., the item about fears based on social identity), the author generated this item directly and aligned its wording with the structure of the existing item pool. See supplemental materials.

The first version of the scale contained 24 items, half of which were directly modified from the IBQ and IBQ.2, and the other half were derived from other literature reviewed above. Eight items were reverse coded. In reflection of the original dimensions described by Smith and Freyd (2014), fear of institutional betrayal maps onto the omission-commission and isolated-systemic continuums by capturing expectations of how institutional harm may manifest across contexts. Specifically, items on the FIBQ reflect concerns about active institutional harm (e.g., being slow to respond to reports) and passive failures of response (e.g., not taking reports seriously), as well as one-on-one concerns (e.g., fear of being blamed) and systemic problems (e.g., covering up sexual violence).

## Study 1 Rationale

This study focused on the experiences of women and gender minorities, which is consistent with other research on institutional betrayal in similar contexts that have used predominantly women samples (e.g., Lind et al., 2020; Monteith et al., 2021; Reffi et al., 2021; Smith & Freyd, 2013, p. 2017), and reflects the fact that women and gender minorities are disproportionately affected by gender-based and sexual violence compared to their male counterparts, particularly in post-secondary settings (Fedina et al., 2018). This focus also addresses the structural imbalance in gender socialization norms in institutional contexts (Gardiner & Finn, 2022). That is, risk of violence and risk of poor institutional responses are components of social discourse that women experience as members of society and of respective institutions (Gray, 2024; Phipps, 2020; Riley, 2019). Understood in the context of gendered social norms and rape culture, the construct of fear of institutional betrayal is inherently aligned with other feminist scholarship exploring gendered institutional harm, especially in neoliberal universities, and the way that educational institutions sustain gender inequality through everyday practices (Gardiner & Finn, 2022; Phipps, 2020). Fear of institutional betrayal is an anticipatory fear, rooted in social knowledge that institutional harm against women has happened before, and continues to affect survivors of gender-based violence (Gray, 2024; Smidt et al., 2023). Accordingly, situating women's experiences of fear of institutional betrayal contributes to academic discourse on gender as a consideration in institutional credibility narratives (Gardiner & Finn, 2022; Riley, 2019).

As such, the current research was purposely focused on the population for whom sexual violence and institutional betrayal are most prevalent, socially salient, and empirically documented: women in post-secondary settings. This target demographic produced a more homogeneous sample than one that included men, allowing clearer interpretation of the scale's initial reliability and validity. This recruitment approach avoided mixing the potentially different experiences men and women may have with sexual violence and institutional betrayal, including varied perceptions of institutional supportiveness based on gender. Research on optimism bias suggests men are typically less likely to assume they will experience sexual violence victimization (Chapin & Pierce, 2012; Lane et al., 2009), suggesting men may be less likely to believe that institutional betrayal could happen to them as well. Differential perceptions of institutional betrayal by gender may complicate the factor structure and warrant distinct validation analyses. Once the construct is more fully established, the scale can be extended to include a broader range of experiences and validated in broader student populations, allowing for examination of how fear of institutional betrayal may manifest differently among men and women.

This study had three research questions. Research Question 1 was: What are the psychometric properties of the FIBQ? To answer this first question, the goal was to evaluate the factor structure and internal consistency, including establishing evidence for the scale's reliability. Research Question 2 was: How well does the FIBQ demonstrate convergent and divergent validity with other relevant psychological constructs? To answer this question, it was necessary to compare the FIBQ to other established measures. First, i*nstitutional trust* is sometimes discussed in terms of confidence in the university as a broad institution that is capable of helping survivors (Rizzo et al., 2021; Sulkowski, 2011). Institutional trust is not specific to sexual violence response expectations, nor does it reflect perceptions of institutional readiness to handle sexual violence, making it an implicitly broader concept than fear of institutional betrayal. Fear of institutional betrayal also reflects the distinct and explicit fear that harm will come to individuals who report sexual violence to the institution, a dimension that institutional trust does not capture.

Next, *perceptions of procedural justice* reflect individuals’ beliefs about the fairness, transparency, and respectfulness of the processes and decision-making practices used by authorities (Lucas et al., 2011; Tyler, 1998). Procedural justice is achieved when decision-making processes are perceived as fair, regardless of the outcome of the decision (Tyler, 1998). There is generally an association between higher levels of perceived procedural justice and greater levels of institutional trust and satisfaction (Tyler, 1998). Some aspects of fear of institutional betrayal reflect the opposite of this perception. Fear of institutional betrayal includes the concern that procedures are not fair and that institutional processes result in harm. As such, both institutional trust and perceived procedural justice are theoretically negatively related to fear of institutional betrayal but are not identical, making them relevant constructs for assessing the convergent validity of the FIBQ.

Next, this study used *just world beliefs* and *generalized anxiety* to assess the discriminant validity of the FIBQ. Just world beliefs (Lerner & Simmons, 1966) are a cognitive bias centered on the idea that people “get what they deserve” and that, because the world is just and fair, misfortune results from poor individual choices (Lipkus, 1991). This belief should be different from fear of institutional betrayal, given that fear of institutional betrayal reflects anticipation of harm regardless of the presumed “deservedness” of the survivor. Fear of institutional betrayal is unrelated to choice.

Generalized anxiety refers to the experience of frequent and persistent worry, nervousness, and tension that is difficult to control (Löwe et al., 2008). Reffi and colleagues (2021) reported that scores on the IBQ.2 were unrelated to measures of anxiety. Although anxiety is indicated by feelings of nervousness or worry, fear of IB is distinct in that it specifically and exclusively applies to the fear that the institution will respond negatively to campus sexual violence. Therefore, it was hypothesized that the FIBQ would be strongly negatively associated with institutional trust and perceptions of procedural justice, and weakly associated (or not associated at all) with just world beliefs and generalized anxiety.

Finally, Research Question 3 was: Are there any group-based differences in fear of institutional betrayal scores based on demographic characteristics, such as race/ethnicity, sexuality, disability status, sexual violence victimization history, or others? Victimization history could potentially affect fear of institutional betrayal levels given that there is past research that suggests students who have experienced sexual violence report less trust in their institution's response than those who have not (Holland, 2020). Past research has found that student survivors often report feeling uncertain and distrustful of institutions such as the medical, criminal justice, and educational systems (Hackman et al., 2022; Smith, 2017), and fear future negative treatment (Ahern, 2018; Reffi et al., 2021). It was therefore hypothesized that fear of institutional betrayal may differ across demographic characteristics, such as sexual violence victimization, but given the fact that fear of institutional betrayal is a new contribution to the literature, and the exploratory nature of these analyses, these hypotheses were not directional.

## Study 1 Method

### Procedure

Participants were recruited through the University of Windsor Psychology Participant Pool in the Winter semester of 2025. Clearance was obtained from the university's Research Ethics Board. Participants who identified as women or gender minorities (e.g., non-binary, two-spirit) and were 16 years of age or older were eligible to participate. In accordance with the Canadian Tri-Council Policy Statement: Ethical Conduct for Research Involving Humans and institutional Research Ethics Board approval, all participants provided their own informed consent to participate. Participants completed a 10minute survey that included a demographics questionnaire, the FIBQ, and a series of other related questionnaires presented in randomized order. Participants then completed the Sexual Experiences Survey-Short Form Victimization (SES-SFV) to measure victimization history. At the end of the survey, participants received a feedback letter with a national list of community mental health and sexual violence-related resources. Participants were compensated with psychology bonus points through the participant pool.

## Participants

A total of 327 women or gender-minority students opened the survey. Of these, 17 people were removed for the following reasons: non-consent (*n* = 1), did not answer any questions (*n* = 3), poor data quality (e.g., speeders; *n* = 6), and incomplete responses (*n* = 7), leaving a final sample of 310 participants. This sample size was more than sufficient for testing a scale of this length, consistent with current best practices in scale development (Floyd & Widaman, 1995; DeVellis & Thorpe, 2021). Full demographic details are available in Table 1. Participants in this study were between the ages of 17 and 42 (*M* = 20.79, *SD* = 2.92). Most participants identified as women (*n* = 305, 98%). The sample was predominantly White (*n* = 179, 57.7%) and heterosexual (*n* = 232, 74.5%). Participants represented several academic areas, including the sciences, social sciences, education, humanities, and social work programs.

**Table 1.** Demographic Characteristics of Participants in Studies 1 and 2.

		Study 1 (*n* = 310) *n* (%)	Study 2 (*n* = 412) *n* (%)
If you had to select ONE response that best describes your current gender identity, what would it be?			
	Female	305 (98.4)	412 (100)
	Non-binary, genderqueer, agender, or a similar gender identity	5 (1.6)	N/A^a^
Do you identify as transgender?			
	Yes	3 (1.0)	3 (.7)
	No	306 (98.7)	406 (98.7)
	Prefer not to say	1 (.3)	3 (.7)
Province of post-secondary Institution			
	British Columbia	N/A	52 (12.6)
	Alberta	N/A	53 (12.9)
	Saskatchewan	N/A	7 (1.7)
	Manitoba	N/A	15 (3.6)
	Ontario	310 (100)^b^	219 (53.2)
	Quebec	N/A	36 (8.7)
	New Brunswick	N/A	2 (.5)
	Nova Scotia	N/A	9 (2.2)
	Prince Edward Island	N/A	2 (.5)
	Newfoundland and Labrador	N/A	6 (1.5)
	Online program or not specified	N/A	11 (2.7)
University or college/Trade school			
	University	310 (100)	267 (64.8)
	College/Trade school	N/A	145 (35.2)
What is your year of study?			
	First-year undergraduate	39 (12.6)	126 (30.6)
	Second-year undergraduate	79 (25.5)	110 (26.7)
	Third-year undergraduate	104 (33.5)	62 (15.0)
	Fourth-year undergraduate	69 (22.3)	42 (10.2)
	Fifth-year undergraduate or higher	19 (6.1)	19 (3.2)
	Other (e.g., trade or vocational student, graduate student, law, medicine)	0 (0)	59 (14.3)
What is your sexual identity/orientation?			
	Asexual	12 (3.9)	14 (3.4)
	Bisexual or pansexual	38 (12.3)	68 (16.5)
	Gay / lesbian	7 (2.3)	11 (2.7)
	Heterosexual / Straight	232 (74.8)	289 (70.1)
	Queer	13 (4.2)	9 (2.2)
	Another sexual identity	0 (0)	3 (.7)
	Prefer not to say or did not respond	8 (2.6)	18 (4.4)
Which of the following best describes your ethnic background?			
	White, European, or European-Canadian	179 (57.7)	168 (40.8)
	First Nations, Indigenous, Inuit, or Métis	4 (1.3)	10 (2.4)
	Black, African, Caribbean, African Canadian, or Caribbean Canadian	16 (5.2)	71 (17.2)
	East Asian, Pacific Islander, East Asian Canadian, or Pacific Islander Canadian	4 (1.3)	27 (6.6)
	South Asian, Southeast Asian, South Asian Canadian, or Southeast Asian Canadian	28 (9.0)	64 (15.5)
	Middle Eastern, North African, Middle Eastern Canadian, or North African Canadian	54 (17.4)	26 (6.3)
	Latin, Central American, South American, Latin Canadian, Central American Canadian, or South American Canadian	7 (2.3)	12 (2.9)
	Bi- or multi-racial	15 (4.8)	19 (4.6)
	Another ethnicity not listed above	3 (1.0)	7 (1.7)
	Prefer not to say or did not respond	0 (0)	8 (2.0)
Do you identify as Indigenous (status or non-status)?			
	Yes	10 (3.2)	24 (5.8)
	No	300 (96.8)	388 (94.2)
Do you identify as an ethnic or racial minority?			
	Yes	99 (31.9)	161 (39.1)
	No	209 (67.4)	251 (60.9)
	Did not respond	2 (.6)	0 (0)
Do you have a disability?			
	Yes	36 (11.6)	73 (17.7)
	No	273 (88.1)	338 (82.0)
	Did not respond	1 (.3)	1 (.2)
Are you registered as a mature student?^c^			
	Yes	35 (11.3)	112 (27.2)
	No	272 (87.7)	300 (72.8)
	Did not respond	3 (1.0)	0 (0)
Are you registered as a part-time student?			
	Yes	21 (6.8)	66 (16.0)
	No	288 (92.9)	345 (83.7)
	Did not respond	1 (.3)	1 (.2)
Are you an international student?			
	Yes	16 (5.2)	86 (20.9)
	No	293 (94.5)	325 (78.9)
	Did not respond	1 (.3)	1 (.2)
Did you move away from home to attend university?			
	Yes	98 (31.6)	218 (52.9)
	No	212 (68.4)	194 (47.1)
Did one or both of your parents complete post-secondary education (e.g., received a university degree or college diploma)?			
	Yes	237 (76.5)	294 (71.4)
	No	65 (21.0)	97 (23.5)
	Did not respond	7 (2.6)	21 (5.1)

*Note*. Some percentages may not total to 100 due to rounding.

a All participants in Study 2 identified as women because that was one of the eligibility criteria. Qualtrics Panels were not able to recruit non-binary participants for this study.

b All participants in Study 1 were recruited from the same institution in southwestern Ontario.

c A mature student is a Canadian citizen or permanent resident who was at least 20 years old and had not attended secondary school full-time in the 2 years prior to beginning their program.

## Measures

### Demographics

The first section of the survey included a brief demographics questionnaire that inquired about age, gender, racial/ethnic background, student status (i.e., international student, mature student [see note in Table 1], part-time student), sexuality, and disability status. Participants were also asked if they had moved away from home to attend university; this question identified students whose familiar healthcare providers, social support systems, and connections to more traditional/spiritual support sources might have no longer been accessible to them.

### Fear of Institutional Betrayal Questionnaire

Given that the FIBQ is an attitudinal scale, participants in this study indicated their level of agreement with each statement using the following 7-point Likert scale: (1) *Strongly Disagree,* (2) *Disagree,* (3) *Somewhat Disagree,* (4) *Neither Agree nor Disagree,* (5) *Somewhat Agree,*(6) *Agree*, (7) *Strongly Agree.*

### Institutional Trust

The Trust in Campus Supports Scale (Sulkowski, 2011) is a 6-item measure designed to assess respondents’ degree of trust in campus-based support resources. Participants responded to items on this scale using a 5-point Likert scale ranging from 1 (*strongly disagree*) to 5 (*strongly agree*). In this study, the items were averaged to create a mean score with higher scores indicating more positive perceptions of the institution's ability to provide support to students (Herres et al., 2021). An example item includes, “There is a good support system on campus for students going through difficult times.” In the original study, Sulkowski (2011) reported a unidimensional factor structure with good internal consistency (Cronbach's alpha = .83) and established convergent and discriminant validity with other constructs (e.g., campus connectedness and fear of negative evaluation, respectively, Sulkowski, 2011). The scale has also performed well in other research. Nightingale (2022) reported an alpha of .81, and Herres et al. (2021) reported an alpha of .84. In the current study, the scale demonstrated acceptable reliability (see benchmarks from Nunnally, 1978) (α = .75).

### Perceived Procedural Justice

In the current study, to ensure the measure of perceived procedural justice was relevant to the post-secondary context, these attitudes were measured using a method modeled after Rizzo and colleagues (2021). Rizzo et al. (2021) modified a set of items from previous studies on procedural justice in other settings (see Folger & Konovsky, 1989; & Reisig & Lloyd, 2009; as cited in Rizzo et al., 2021). This method was chosen because it allowed participants to assess their perceptions of procedural justice on campus, even if they had not personally experienced sexual violence victimization. Participants rated their agreement with statements assessing perceptions of procedural justice regarding campus police and university administration (respectively) on 5-point Likert scales (1—*Strongly Disagree* to 5—*Strongly Agree*). An example item includes, “Campus police are completely candid and frank when interacting with students.” Separate mean scores were computed for perceptions of campus police and university administration. In Rizzo et al.’s (2021) study, both subscales demonstrated excellent reliability (α = .92 for both), although they did not explore convergent or divergent validity for the scales. In the present study, reliability was good for perceptions of campus police (α = .81) and university administration (α = .89). An overall procedural justice score, calculated by averaging all items, also demonstrated excellent reliability (α = .92).

### Just World Beliefs

The Global Belief in a Just World Scale (Lipkus, 1991) is a 7-item questionnaire that assesses overall beliefs in a just world. Items (e.g., I feel that people get what they are entitled to have”) were rated on a 1 (*strongly disagree*) to 6 (*strongly agree*) scale and summed to create an overall measure of just world beliefs. The original study reported a unidimensional factor structure, acceptable reliability, as well as convergent validity (e.g., with locus of control and perceived sincerity) and divergent validity (e.g., cautiousness, perceived control based on chance). A review by Hellman et al. (2008) indicated the Global Belief in a Just World Scale demonstrates acceptable internal consistency and reliability compared to other versions, and other scholarship on just world belief theory indicates general support for the use of the Global Belief in a Just World Scale (Hafer & Sutton, 2016). This scale is widely used in psychology (e.g., Zhang & Zhang, 2015) and, specifically, in research on sexual violence (e.g., Cherniawsky & Morrison, 2022; Hayes et al., 2013), and thus was chosen for this study. The scale demonstrated acceptable reliability in the current study (α = .79).

### Generalized Anxiety

The Generalized Anxiety Disorder Screener (often referred to as the GAD-7; Löwe et al., 2008; Spitzer et al., 2006) is a 7-item report measure that asks respondents to reflect on and report the frequency of anxiety symptoms (e.g., irritability) on a 4-point scale, ranging from 0 (*not at all*) to 3 (*nearly every day*). Items are summed, with higher scores indicating more frequent experiences of anxiety. The Generalized Anxiety Disorder Screener is considered one of the gold standard brief tools for assessing anxiety (Plummer et al., 2016). This tool has been tested and validated in a range of clinical and general population samples (Löwe et al., 2008) and has been used in other studies on campus sexual violence and institutional betrayal (e.g., Lett et al., 2020). The scale is validated and is supported for use with post-secondary samples and has shown excellent reliability when used with this population (e.g., see validation study by White & Karr, 2025, who reported an alpha of .91). The scale demonstrated evidence of excellent reliability in the current study (α = .92).

### Sexual Victimization

Measuring sexual violence victimization is necessary for group-based difference tests of FIBQ scores based on victimization history. The Sexual Experiences Survey-Short Form Victimizationn (SES-SFV, Koss et al., 2007) is considered the gold standard and most used measure of sexual victimization experiences (e.g., Fedina et al., 2018; Sall & Littleton, 2022). A study by Johnson et al. (2017) reported that the scale demonstrated strong internal consistency (around .92) and good test–retest reliability. They also reported that it demonstrated construct and predictive validity, as victimization scores were associated with trauma symptoms (e.g., anxiety, depression, dissociation). The measure uses behaviorally specific language in place of terms such as “sexual assault,” “sexual violence,” or “rape,” and is commonly used in research on campus sexual violence (e.g., Mellins et al., 2017; Neutzling et al., 2024; Sall & Littleton, 2022; Senn et al., 2015).

The SES-SFV contains a list of seven behaviorally specific actions (e.g., “A man put his penis into my vagina, or someone inserted fingers or objects without my consent”). For each behavior, respondents are asked to review a list of coercive tactics and indicate whether that has happened to them since age 14 (e.g., “by threatening to physically harm me or someone close to me”). This study used non-redundant severity scoring (Davis et al., 2014; Koss et al., 2007), which allows for the classification of respondents into a category based on the severity of their experiences (e.g., attempted rape versus rape). Specifically, participants were scored as either non-victims (if they endorsed no items on the scale) or having experienced one of five categories of victimization, based on the most severe items endorsed: unwanted sexual experiences, attempted coercion, coercion, attempted rape, or rape. Participants who were scored as non-victims were compared to participants who had experienced any other form of sexual violence. Follow-up analyses examined differences by victimization type.

## Data Analysis

Analyses for this study were conducted using SPSS version 30 for Mac. Previous research has yielded mixed results about the factor structure of the original IBQ (Reffi et al., 2021; Smith & Freyd, 2013). Given the past mixed results and the fact that *fear* of institutional betrayal is a new conceptual application and extension of the institutional betrayal framework, exploratory methods were appropriate. Thus, to answer Research Question 1, the first step of analysis was exploratory factor analysis (Field, 2018; Floyd & Widaman, 1995). Factor retention was determined using a combination of eigenvalues greater than 1, inspection of the scree plot, and theoretical interpretability of the factor structure. Items were retained if they demonstrated primary factor loadings of at least .40. Inter-item correlations were examined to ensure items were meaningfully related (i.e., low to moderate correlations, ideally ∼.30 or .40), but not redundant (i.e., greater than .80). Reliability analysis (i.e., Cronbach's alpha) was used to explore item-if-removed coefficients (which informed decisions about item removal), and to ultimately determine the internal consistency of the final FIBQ. To validate the factor structures, Velicer's minimum average partial (MAP) tests (using the Revised 2000 Test scores, see O’connor, 2000) as well as parallel tests were conducted. To answer Research Question 2 and establish evidence of construct validity, Pearson's correlations were used to examine relations between fear of institutional betrayal and related psychological constructs. Finally, to answer Research Question 3, independent-samples *t*-tests and analysis of variance (ANOVA) were used to explore group-based differences in fear of institutional betrayal, such as by sexual violence victimization history, sexuality, and student status.

## Study 1 Results and Discussion

### Factor Structure, Item Analysis, and Reliability Analysis

Statistical assumptions were examined to ensure factorability, absence of multicollinearity, and absence of redundancy in the inter-item correlations. Bartlett's test of sphericity was significant, χ^2^(276) = 4350.98, *p* < .001, and the Kaiser—Meyer—Olkin (KMO) measure of sampling adequacy was excellent (KMO = .95). Subsequent tests indicated that the item set was tightly structured, with all items sufficiently related to each other while remaining sufficiently distinct to support factor analysis (Pearson's *r*'s ranged from .20 to .70, all *p*'s < .001).

To answer Research Question 1, principal axis factoring (PAF) with a direct oblimin rotation (Field, 2018) was conducted. The scree plot revealed a clear inflection point after the first factor. The eigenvalues for a second factor were marginally above 1. Inspection suggested that this was primarily due to the reverse-coded items clustering together, suggesting a potential methodological artifact. However, the potential second factor accounted for only 4% of the variance, and the steep drop-off combined with the visual elbow supported a one-factor solution. The one-factor model accounted for 46.52% of the total variance. Next, to further evaluate the factor structure, a MAP test and a parallel test were conducted (O’connor, 2000). First, the smallest average squared partial correlation was .0141, and the smallest average fourth power partial correlation was .0006, which were achieved when two components were partialled out, suggesting a two-component solution. The parallel analysis was conducted using 95th percentile eigenvalues derived from randomly generated datasets. Results indicated that two eigenvalues from the observed data exceeded those obtained from random data. The eigenvalue from the first factor in the data (11.16) well exceeded that of the random data (1.63) However, the second eigenvalue (1.555) only marginally exceeded the corresponding random eigenvalue (1.515). Most importantly, in both tests, inspection of the item content indicated that this second component reflected clustering of reverse-coded items rather than a substantively meaningful factor. On this basis, the FIBQ was treated as unidimensional in the present sample. However, the issues with the reverse-coded items were noted. To further evaluate whether the observed pattern reflected true multi-dimensionality or simply methodological artifacts of item wording, alternative non-reverse-coded versions of these items were included in Study 2.

Factor loadings for the single-factor solution ranged from .45 to .78, indicating that all items demonstrated moderate to strong association with the extracted factor (fear of institutional betrayal). The lowest item loading was item 13, “Officials (e.g., administrators) at my university should do more to protect students from sexual violence” (λ = .45). Analysis of the inter-item correlation matrix similarly indicated that no items failed to significantly correlate with the rest of the scale. Item 13 had consistently weaker correlation coefficients (λ range = .21 to .38), but they remained statistically significant. A reliability analysis was conducted on the full 24-item scale. The FIBQ demonstrated excellent internal consistency (α = .948). Item-total statistics indicated that no single item substantially improved reliability if removed, with all “alpha if item deleted” values remaining within .003 of the total alpha. Removal of item 13 would have raised the alpha to .949, which was not deemed a substantial enough change to warrant item deletion. Thus, no items were removed based on loading strength, lack of correlation, or reliability statistics. These results provide preliminary evidence that responses to the FIBQ were consistent.

The overall mean score of the FIBQ was 3.01 (*N* = 310, *SD* = 1.29, possible range = 1–7, observed range 1.00–5.96), with a median of 3.00. While the Kolmogorov—Smirnov (*D* = .05, *p* = .04) and Shapiro—Wilk (*W* = .99, *p* = .03) tests indicated statistically significant deviations from normality, these results are likely inflated due to the sample size (*N* = 310). Skewness (.23, *SE* = .14) and kurtosis (−.07, *SE* = .28) were both within acceptable limits, suggesting approximate normality. This conclusion was supported by visual inspection of the histogram and Q—Q plot, which revealed a roughly normal distribution, further supporting the assumption of normality for the FIBQ scale.

## Assessing the Validity of the Fear of Institutional Betrayal Questionnaire

To answer Research Question 2, and following best practice recommendations (Clark & Watson, 1995; DeVellis & Thorpe, 2021), concurrent predictive validity was assessed by correlating fear of IB with related constructs (expecting moderate associations, *r* ≈ .40 to .70), and discriminant validity by examining correlations with discriminant constructs (expecting weak associations, *r* < .30). As hypothesized, the FIBQ was strongly negatively correlated with institutional trust as measured by the Trust in Campus Supports Scale: *r*(310) = −.74, *p* < .001, consistent with the conceptual relationship between these constructs. Although the correlation coefficient was above .70, this is not an unreasonably strong relation, especially given that some FIBQ items were conceptually modeled after items from the Trust in Campus Support Scale. However, the item content is not unique to the Trust in Campus Supports Scale; rather, it reflects themes and wording commonly found across broader literatures on institutional betrayal, institutional trust, and campus responses to violence. Nevertheless, the adaptation of similarly worded items from the Trust in Campus Supports Scale to the FIBQ may explain the strong correlation. Yet, the scales are clearly not conceptually identical (*r* < .80).

To further evaluate the construct uniqueness of the FIBQ relative to the Trust in Campus Supports Scale, an exploratory factor analysis was conducted that included items from both scales. PAF with a direct oblimin rotation was used. Based on the eigenvalue criterion and assessment of the scree plot, results supported a two-factor solution: The first factor explained 42.9% of the variance and was defined primarily by the FIBQ items, with loadings ranging from .21 to .83. Factor 2 accounted for a further 5.76% of the variance and was defined primarily by the items from the Trust in Campus Supports Scale, with loadings ranging from .29 to 68. Some items from either scale demonstrated modest cross-loadings (< .40). However, as noted, this was expected given the similar wording of some items, and the majority of items loaded strongly and cleanly onto their respective factors. The analysis was repeated using maximum likelihood (ML) extraction with oblique rotation. The ML results produced the same two-factor solution with minor cross-loadings, again distinguishing the FIBQ items from the Trust in Campus Supports Scale items. The replication of the two-factor structure across both PAF and ML extractions provides strong evidence that the FIBQ represents a distinct but related construct from institutional trust, rather than an artifact of the extraction method. These results support construct validity and indicate that fear of institutional betrayal is not redundant with institutional trust.

Next, as hypothesized, the FIBQ was strongly negatively correlated with students’ perceptions of procedural justice, *r*(310) = −.62, *p* < .001. A factor analysis using PAF (direct oblimin rotation) strongly supported this finding. Eigenvalues and examination of the scree plot indicated a very clear two-factor solution in which the FIBQ items cleanly loaded onto factor one (36.1% of variance explained) and perceptions of procedure justice items loaded onto factor two (an additional 9.0% of variance explained). Correlations between the FIBQ and each of the procedural justice subscales (perceptions of campus police and perceptions of campus administration) were also statistically significant: *r*(310) = −.57, *p* < .001; *r*(310) = −.55, *p* < .001, respectively.

It was hypothesized that the FIBQ would not be significantly correlated with just world beliefs, given that the two constructs are theoretically distinct. Results suggested a small but statistically significant negative correlation between the FIBQ and The Global Belief in a Just World Scale, *r*(310) = −.31, *p* < .001. Although the correlation reached statistical significance, the sample size is considerable (*N* = 310) and the effect size was modest and close to the conventional cutoff for a “small” association (< .30; Cohen, 1988), although it is worth noting that some more recent authors have argued that a coefficient of .20 or higher could be considered moderate (Gignac & Szodorai, 2016). Nevertheless, taken together, these results suggest that the FIBQ and just world beliefs represent conceptually distinct but potentially related constructs, consistent with the expectation of discriminant validity. Further validation and replication were warranted in Study 2 to confirm the independence of these measures. Finally, it was hypothesized that the FIBQ would not be significantly correlated with generalized anxiety symptoms as measured by an anxiety screening scale. Results indicated a very small but statistically significant positive correlation between fear of institutional betrayal and anxiety: *r*(310) = .19, *p* = .001. Although significant, the association was minimal, consistent with the interpretation that fear of institutional betrayal and anxiety are distinct constructs.

## Group Difference Tests

To answer Research Question 3 and to further explore the construct validity of the FIBQ when cell sizes were sufficient (i.e., *n* ≥ 30 per group), group-based comparison tests were run to determine whether fear of institutional betrayal scores varied across student groups. First, age was not significantly correlated to fear of institutional betrayal (*r* = .05, *p* = .37). Next, participants who identified as a racial or ethnic minority (*n* = 99, *M* = 3.42, *SD* = 0.94) reported significantly higher fear of institutional betrayal than those who indicated they were not a minority (*n* = 209, *M* = 2.95, *SD* = 2.94), *t*(306) = 4.24, *p* < .001, with a strong effect size (Cohen's *d* = .52). Participants who identified as having a disability (*n* = 36, *M* = 3.47, *SD* = 1.06) reported significantly higher fear of institutional betrayal than those who indicated they did not have a disability (*n* = 273, *M* = 3.05, *SD* = .94), *t*(307) = 2.51, *p* = .01, with a moderate effect size (Cohen's *d* = .45).

An independent-samples *t*-test was conducted to examine whether FIBQ scores differed by sexuality. In order to obtain cell sizes sufficiently powered for analysis, queer-identified students (i.e., those who identified their sexuality as gay, lesbian, bisexual, pansexual, asexual, or queer) were categorized in one group (“queer-identified students”; *n* = 70) and were compared to students who identified as heterosexual (“straight”). Queer-identified students (*M* = 3.38, *SD* = 0.99) reported significantly higher fear of institutional betrayal than those who identified as straight (*n* = 232, *M* = 3.03, *SD* = 0.92), *t*(300) = −2.75, *p* = .006, with a moderate effect size (Cohen's *d* = −.38).

Tests were also conducted to explore the relationship between the length of time in university and fear of institutional betrayal. A one-way ANOVA based on the number of years registered was statistically significant, *F*(3, 287) = 3.06, *p* = .029, η^2^ = .03, including with Robust tests: Welch's *F*(3, 130.22) = 3.91, *p* = .01. Tukey post-hoc tests revealed that first-year students (*M* = 2.72, *SD* = .77) reported significantly lower fear of institutional betrayal than second-year students (*M* = 3.25, *SD* = 1.03), *p* = .02, and fourth- or fifth-year students (*M* = 3.21, *SD* = 1.01), *p* = .048. No other pairwise comparisons were significant. There were no significant differences based on mature student status (*t*[305] = .46, *p* = .65, Cohen's *d* = .08), students who moved away from home (or did not) to attend university (*t*[308] = .81, *p* = .42, Cohen's *d* = .10), or first-generation student status (*t*[300] = −.504, *p* = .61, Cohen's *d* = −.07).

Finally, tests were conducted to examine whether fear of institutional betrayal differed based on participants’ sexual violence victimization history, as assessed by the SES-SFV. Approximately half (49.4%) of participants reported experiencing one or more types of victimization since age 14. A total of 62 out of 310 participants in this study reported experiences of rape (20.0%). Further, 23.2% of participants reported experiences of attempted rape, 28.4% reported experiences of coercion, 31.0% reported experiences of attempted coercion, and 43.5% reported experiences of unwanted sexual contact. Overall, victims of sexual violence (*n* = 153, *M* = 3.21, *SD* = .98) reported significantly higher fear of institutional betrayal than non-victims (*n* = 157, *M* = 2.99, *SD* = .94), *t*(308) = −1.99, *p* = .048, Cohen's *d* = −.23). Next, based on non-redundant severity scoring (Koss et al., 2007), participants were categorized by the most severe form of sexual victimization reported: 50.6% were classified as non-victims, 7.4% sexual contact, 5.2% attempted coercion, 10.6% coercion, 6.1% attempted rape, and 20.0% completed rape. An omnibus ANOVA was statistically significant, *F*(5, 304) = 2.58, *p* = .03, η^2^ = .04. Robust tests also supported this conclusion: Welch's *F*(5, 64.54) = 2.50, *p* = .04. However, Games–Howell post-hoc comparisons revealed that none of the pairwise comparisons reached significance, and no other follow-up tests based on sexual violence victimization were significant (all *p*s > .05).

## Study 2 Rationale

The goal of Study 2 was to replicate and validate the results from Study 1 using a larger, more diverse national sample of post-secondary women across Canada. In Study 1, evaluation of the factor analysis supported a unidimensional factor structure. However, the results also indicated the possibility of a second factor, composed solely of the eight reverse-coded items on the scale, signaling a potential methodological concern. Given this result, in Study 2, participants also responded to duplicate but positively worded versions of the problematic reverse-coded items. For instance, instead of “I believe my university takes reports of sexual violence seriously” (a reverse-coded item), the new item was “I believe my university doesn’t take reports of sexual violence seriously.” Thus, in Study 2, participants completed the original version of the FIBQ exactly as it was completed in Study 1, but they also completed the eight new positively worded versions, which were randomly inserted into the existing questionnaire. Data collection for Study 2 followed that of Study 1 (Winter, 2025).

## Study 2 Methods

### Procedure and Measures

Participants were recruited via a paid Qualtrics Panel through QualtricsXM, an experience management company that offers cloud-based survey and recruitment panels. To be eligible, participants needed to be aged 18+, identify as women, and registered in a post-secondary institution in Canada at the time of the survey. Panel members were sent an email invitation or prompted on the respective survey platform to proceed with the survey.

The survey materials and procedure for this study mirrored those of Study 1. Participants completed a demographics questionnaire, the FIBQ, a measure of institutional trust (α = .77 in the current study), a measure of perceived procedural justice (α = .91), a measure of just world beliefs (α = .83), and a measure of sexual violence victimization (the SES-SFV). Two attention checks were also added to this survey. Participants also completed several other measures outside the scope of the current study. For this reason, and given survey length constraints, the Generalized Anxiety Disorder Screener was not included in this study. Average survey completion time was 15 minutes. At the end of the survey, participants received a feedback letter with a national list of mental health and sexual violence resources. Qualtrics panellists joined from a variety of sources, and participants were compensated through Qualtrics Panels’ standard incentive system. Compensation type and amount (e.g., air miles, reward points, or gift cards) varied across participants and were determined and communicated by Qualtrics prior to survey participation.

## Participants

The survey received 1332 link clicks. Of these, 920 responses were removed: ineligible due to age, gender, or no post-secondary enrollment = 348; non-consent = 74; duplicate responses or bots = 198; poor quality responses (e.g., nonsensical responding, speeders, failed attention checks = 221), and incomplete survey responses = 79. The final sample included 412 participants, which was more than sufficient for the analyses used in this study (DeVellis & Thorpe, 2021). Participants were women aged 18 + who were (at the time of the study) currently registered in a post-secondary institution in Canada. Full demographic details are available in Table 1. Participants in this study ranged in age from 18 to 56 (*M* = 23.96, *SD* = 6.99). All participants identified as women. The sample was relatively diverse in terms of ethnic background. Less than half (*n* = 168; 40.8%) of participants identified as White. Most of the women in the sample identified as heterosexual (*n* = 289; 70.1%). The sample included participants from all 10 Canadian provinces. Unlike many studies focused on post-secondary students which exclusively explore the perspectives of university students, approximately one third of the sample were from colleges or trade schools (*n* = 145; 35.2%). Students represented a diverse range of academic and professional programs across the arts, sciences, engineering, social sciences, humanities, education, business, technology, law, healthcare, and skilled trades.

## Data Analysis

Given that Study 1 indicated a unidimensional factor structure, exploratory factor analysis using PAF with a direct oblimin rotation was used rather than confirmatory factor analysis. The analysis was performed twice: once to replicate Study 1 using the original scale and once using the revised scale (i.e., after removing problematic reverse-coded items and replacing them with positively worded versions). The variation and performance of each item in the scale were evaluated to inform decisions about potential item removal. All subsequent analytic steps were consistent with the procedures described in Study 1.

## Study 2 Results and Discussion

### Factor Structure, Item Analysis, and Reliability Analysis

All statistical assumptions for factor analysis were met, as indicated by a significant Bartlett's test of sphericity (χ^2^[276] = 6793.05, *p* < .001) and an excellent KMO value (KMO = .96). Similar to Study 1, the inter-item correlation coefficients were acceptable (Pearson's *r*'s ranged from .14 to .74, all *p*'s < .001). The scree plot revealed a sharp drop after the first factor, and a second inflection point after the second factor. Eigenvalues indicated a second factor was present. The first factor accounted for 49.7% of the variance, and the second factor accounted for a further 10.0%. Examination of the factor structures indicated that all eight negatively worded items had loaded onto their own factor, suggesting a potential methodological artifact in the data.

Like Study 1, a MAP test and a parallel test were conducted to validate the factor structure determinations (O’connor, 2000). First, results from the MAP test indicated that the smallest average squared partial correlation was .0110, and the smallest average fourth power partial correlation was .0004, which was achieved when two components were partialled out, suggesting a two-component solution. The parallel analysis was conducted using 95th percentile eigenvalues derived from randomly generated datasets. Results indicated that two eigenvalues from the observed data exceeded those obtained from random data. The second eigenvalue in the data (2.41) exceeded that of the random data (1.44). As with Study 1, inspection of the item content indicated that this second component reflected clustering of negatively worded items rather than a substantively meaningful factor.

For the second analysis, the negatively worded items were removed from the scale and replaced with the eight replacement items instead. Bartlett's test of sphericity was significant, χ^2^(276) = 7799.78, *p* < .001, and the KMO measure of sampling adequacy was nearly perfect (KMO = .98). Inter-item correlations were acceptable (Pearson's *r*'s ranged from .24 to .77, all *p*'s < .001). The results of the factor analysis clearly supported a unidimensional solution. Examination of the scree plot revealed a clear inflection point after the first factor, and assessment of the Eigenvalues revealed only one factor with a value higher than 1.00. Results from a MAP test indicated that the smallest average squared partial correlation was .0106, and the smallest average fourth power partial correlation was .0003, which was derived when only one component was partialled out, and thus the MAP test supported a one-component solution. The parallel analysis (conducted using 95th percentile eigenvalues derived from randomly generated datasets) strongly suggested a one-factor solution, as only one eigenvalue from the actual data (13.92) exceeded the mean eigenvalues from the random data (1.51). At the second factor level, the random data (1.44) exceeded that of the actual data (.96), providing additional evidence for a unidimensional structure.

The initial unidimensional factor solution accounted for 58.8% of the variance. Factor loadings ranged from .43 to .84, indicating that all items demonstrated moderate to strong association with the extracted factor (fear of institutional betrayal). In replication of Study 1, the item with the lowest factor loading (.43) was “Officials (e.g., administrators) at my university should do more to protect students from sexual violence.” Analysis of the inter-item correlation matrix indicated that, similar to Study 1, this item had consistently weaker (although still significant) correlations with all of the other items than the rest of the inter-item correlations. The second lowest factor loading was .57. A reliability analysis was conducted on the full 24-item scale. The FIBQ demonstrated excellent internal consistency (α = .969). Item-total statistics mostly indicated that all items contributed to the scale. However, removal of the one problematic item (about university officials) improved the alpha to .970. Given the repeated poor performance in this study and the last, as well as concerns regarding its conceptual alignment with the broader construct of fear of IB, this item was ultimately removed from the measure. The rest of the items all performed well. These results suggest that all remaining items contributed meaningfully to the scale in this sample, and that the measure is internally coherent. After removing the one poor-performing item, the factor analysis was repeated. The final unidimensional structure accounted for 60.53% of the variance. The factor loadings ranged from .571 to .835, and inter-item correlations were strong but not redundant, suggesting cohesion of the measure, ranging from *p* = .31 to .73. The final 23-item FIBQ (Table 2) had a Cronbach's alpha of .97.

**Table 2.** Factor Loadings of Final FIBQ Items (α = .97).

**Item**	**Factor Loading**
1. I worry that, in my university, people who report sexual violence are sometimes punished or experience other negative consequences.	0.57
2. I believe my university doesn’t take reports of sexual violence seriously.	0.77
3. I worry my university creates an environment in which sexual violence seems common or normal.	0.73
4. I’m afraid that victims/survivors of sexual violence aren’t treated with dignity and respect by my university.	0.75
5. I worry that my university does not care about sexual violence.	0.81
6. I’m afraid that when sexual violence is reported, my university is only concerned with protecting its reputation.	0.83
7. I believe that when sexual violence is reported, my university sometimes mishandles cases.	0.75
8. I believe that university administrators deny that sexual violence occurs on campus.	0.74
9. I worry that my university wouldn’t punish someone who engaged in sexual violence on campus.	0.80
10. I’m afraid that people who report sexual violence in my institution won’t be believed.	0.81
11. My university creates an environment in which sexual violence seems more likely to occur.	0.72
12. I worry that my university isn’t taking steps to prevent sexual violence.	0.79
13. I’m afraid that reporting sexual violence might lead to retaliation by my university.	0.82
14. I worry that the university is too slow to respond to incidents of sexual violence on campus.	0.77
15. I worry that my university wouldn’t maintain the privacy of people who report sexual violence.	0.72
16. I believe that my university sometimes works to cover up instances of sexual violence on campus.	0.72
17. I am afraid that people who report sexual violence on campus are labeled as troublemakers.	0.79
18. I worry that victims/survivors who report sexual violence in my university will be blamed.	0.84
19. I worry that my university doesn’t take action to address specific factors that may lead to sexual violence.	0.82
20. I worry that my university would make it difficult or complicated to report sexual violence.	0.82
21. I fear that some people who report sexual violence would receive a poor response from the university because of their identity (e.g., race, ethnicity, sexuality, or gender identity, or pronouns).	0.75
22. I don’t believe my university provides enough support to victims/survivors of sexual violence.	0.74
23. I worry that university representatives (e.g., staff, administrators) probably wouldn’t be able to do anything to help victims/survivors of sexual violence.	0.75

*Note.* FIBQ = Fear of Institutional Betrayal Questionnaire.

Across the sample (*N* = 412), the overall mean score was 3.33 (*SD* = 1.29, possible range = 1–7, observed range 1–7), with a median of 3.43. This suggests that the mean in this sample was slightly elevated compared to the participant pool in Study 1, and that slightly more variation was present, as expected given the diversity of the Study 2 sample. Tests of normality indicated significant deviations from normality (Kolmogorov—Smirnov *D*(412) = .06, *p* = .004; Shapiro—Wilk *W*(412) = .98, *p* < .001). However, these tests are highly sensitive to large sample sizes, and descriptive indices indicated approximate normality, with near-zero skewness (−.01, SE = .12) and acceptable kurtosis (−.68, SE = .24). Visual inspection of the histogram and Q–Q plots confirmed a roughly symmetrical distribution. On this basis, the FIBQ was treated as approximately normally distributed.

## Assessment of the Validity of the Fear of Institutional Betrayal Questionnaire

Replicating Study 1, correlational tests were conducted to establish concurrent and discriminant validity with the three other scales included in the survey. The FIBQ was strongly negatively correlated with the Trust in Campus Supports Scale *(r*[411] = −.61, *p* < .001) and the Perceived Procedural Justice Scale *(r*[412] = −.56, *p* < .001). Correlations between the FIBQ and the procedural justice subscales (perceptions of campus police and perceptions of campus administration) were also statistically significant: *r*(412) = −.506, *p* < .001; *r*(411) = −.52, *p* < .001, respectively. Lastly, the FIBQ was marginally negatively correlated with the just world beliefs, though not nearly enough to signal that the concepts were meaningfully related: *r*(412) = −.10, *p* = 0.04. Together, this evidence replicates the findings from Study 1 and strengthens the evidence that the FIBQ is both a valid and reliable measure.

## Group Difference Tests

Group difference tests (where possible, when cell sizes were sufficient) yielded several significant differences in FIBQ scores by demographics. First, fear of institutional betrayal was significantly negatively correlated with age (*r* = −.15, *p* = .002). Students registered in colleges at the time of the study (*n* = 145, *M* = 3.01, *SD* = 1.32), had significantly lower FIBQ scores than students registered in universities (*n* = 267, *M* = 3.50, *SD* = 1.23), *t*(410) = 3.79, *p* < .001, with a moderate effect size (Cohen's *d* = −.39).

Contrary to Study 1, there was no difference between students who identified as a racial minority (*n* = 262, *M* = 3.42, *SD* = 1.28) and those who did not (*n* = 251, *M* = 3.27, *SD* = 1.29), *t*(410) = 1.15, *p* = .25, Cohen's *d* = −.12). However, replicating Study 1, participants who identified as having a disability (*n* = 73, *M* = 3.77, *SD* = 1.27) reported significantly higher fear of institutional betrayal than those who did not have a disability (*n* = 338, *M* = 3.23, *SD* = 1.25), *t*(409) = 3.27, *p* < .001, Cohen's *d* = .42). Also, in replication of Study 1, queer-identified students (*M* = 105, *SD* = 1.20) reported significantly higher fear of institutional betrayal than those who identified as straight (*n* = 289, *M* = 3.23, *SD* = 1.30), *t*(392) = −2.40, *p* = .02, Cohen's *d* = −.27). Next, there were no significant differences based on mature student status (*t*[410] = 1.07, *p* = .29, Cohen's *d* = −.12), having moved away from home to attend university (*t*[410] = −.53, *p* = .60, Cohen's *d* = −.05), or first-generation student status (*t*[389] = −.77, *p* = .44, Cohen's *d* = −.09).

Tests were also conducted to explore the relation between the length of time in university and fear of institutional betrayal. Similar to Study 1, a one-way ANOVA based on the number of years registered was statistically significant, *F*(4, 392) = 6.96, *p* < .001, η^2^ = .07, including Robust tests: Welch's *F*(4, 144.09) = 7.28, *p* < .001. Tukey post-hoc tests revealed that first-year students (*M* = 2.90, *SD* = 1.16) reported significantly less fear than second-year students (*M* = 3.61, *SD* = 1.24), *p* < .001, third-year students (*M* = 3.67, *SD* = 1.28), *p* < .001, and fourth- or fifth-year students (*M* = 3.55, *SD* = 1.34), *p* = .01. First-year students were not significantly different from graduate students (*M* = 3.15 *SD* = 1.32), *p* = .80. No other pairwise comparisons were significant.

Unlike Study 1, the subgroup sample sizes in Study 2 were sufficient to explore potential differences between international students (compared to domestic students) and between part-time students (compared to full-time students). Results indicated that international students (*n* = 86, *M* = 3.06, *SD* = 1.38) reported significantly lower fear of institutional betrayal than domestic students (*n* = 325, *M* = 3.40, *SD* = 1.25), *t*(409) = −2.15, *p* = .03, Cohen's *d* = −.26). Conversely, there was no difference in fear of institutional betrayal in part-time students (*n* = 66, *M* = 3.51, *SD* = 1.37) compared to full-time students (*n* = 409, *M* = 3.29, *SD* = 1.27), *t*(409) = 1.26, *p* = .21, Cohen's *d* = .17.

Finally, tests were conducted to explore sexual violence victimization and fear of institutional betrayal. More than half (58.3%) of participants reported experiencing one or more types of victimization since age 14. A total of 141 out of 412 participants in this study reported experiences of rape (34.2%) at some point in their lifetime since age 14. Further, 28.4% of participants reported experiences of attempted rape, 37.9% reported experiences of coercion, 35.2% reported experiences of attempted coercion, and 54.1% reported unwanted sexual contact. Overall, there was no significant difference between victims of sexual violence (*n* = 240, *M* = 3.39, *SD* = 1.28) and non-victims regarding fear of institutional betrayal (*n* = 172, *M* = 3.23, *SD* = 1.29), *t*(410) = −1.25, *p* = .21, Cohen's *d* = −1.29. Based on non-redundant severity scoring (Koss et al., 2007), participants were categorized by the most severe form of sexual victimization reported: 41.7% were classified as non-victims, 7.5% sexual contact, 3.9% attempted coercion, 9.2% coercion 3.4% attempted rape, and 34.2% completed rape. Notably, most victims of rape also reported other less severe experiences as well. A one-way ANOVA was conducted to examine potential differences across victimization categories. The omnibus test was not statistically significant, *F*(5, 406) = 1.35, *p* = .24, η^2^ = .02, suggesting that fear of institutional betrayal did not vary across victimization categories. Robust tests also supported this conclusion: Welch's *F*(5, 62.29) = 1.40, *p* = .24. Notably, a follow-up test comparing only victims of rape to non-victims was significant: *t*(311) = −2.06, *p* = .02, Cohen's *d* = 1.23, such that victims of rape (*M* = 3.54, *SD* = 1.31) reported significantly more fear of institutional betrayal than non-victims (*M* = 3.23, *SD* = 1.29).

## General Discussion

Fear of institutional betrayal is a new conceptual extension of the original institutional betrayal framework. The original framework (Smith & Freyd, 2013, 2014) provides researchers and survivors of violence with a way of making sense of, and giving credibility to, the experiences of harm perpetrated by institutions. This body of research clearly demonstrates that experiencing institutional betrayal is associated with a range of adverse consequences for survivors of violence (Christl et al., 2024). Fear of institutional betrayal builds upon this framework, reflecting anticipatory fear of institutional betrayal occurring in the future. The original measure of institutional betrayal, the IBQ (Smith & Freyd, 2013), is a checklist of institutional actions or inactions that perpetuate harm to survivors of sexual violence. Conversely, fear of institutional betrayal is an attitudinal construct. Any member of a community (regardless of victimization history) may fear that, if something bad were to happen to them, institutional betrayal might subsequently follow. Moreover, any individual may harbor concerns about systemic neglect of sexual violence issues on campus. Accordingly, fear of institutional betrayal is the belief that institutions will inevitably fail to prevent or acceptably respond to instances of sexual violence, and that engagement with the institution in the aftermath of sexual violence will most likely result in harm for the survivors.

The concept of fear of institutional betrayal fits within the broader theoretical literatures on institutional responses to gender-based violence (Campbell et al., 2023; Dufour, 2024; Gretgrix & Farmer, 2026; Smidt et al., 2023), help-seeking barriers (Nightingale, 2022; Spencer et al., 2017), campus climate (Kettrey et al., 2026; Lind et al., 2020; Swartout et al., 2019), structural power (Phipps, 2020; Riley, 2019), institutional harm (Phipps, 2020; Smith & Freyd, 2014), gendered social norms (Lewis et al., 2018; Lind et al., 2020; Linder et al., 2019), structural silencing (Gray, 2024; Riley, 2019), perceptions of procedural justice (Lucas et al., 2011; Tyler, 1998), anticipatory fear of crime (Lane et al., 2009), and institutional trust (Nightingale, 2022; Rizzo et al., 2021; Sulkowski, 2011).

## Reliability and Validity of the Fear of Institutional Betrayal Questionnaire

The two presented studies provide evidence of the construct validity and existence of fear of institutional betrayal in post-secondary students. These studies also provide robust evidence for the reliability and validity of the new FIBQ. Study 1 used a participant sample from one university in southwestern Ontario and demonstrated promising results. Study 2 replicated Study 1 using a larger, more diverse sample of women registered at post-secondary institutions across Canada, and allowed the measure to be slightly revised and refined based on psychometric analyses. The result is a unidimensional 23-item measure that uses a 7-point scale. The unidimensional factor structure and high internal consistency (α = .97) provide strong evidence that respondents reliably responded to items assessing fear of institutional betrayal. The descriptive analysis and normality findings suggest that the scale is likely appropriate for parametric analyses in future research.

We note that, unlike in many scale development studies, only one item was dropped from the original version of the scale (in addition to revising the wording of the final tool). The factor loadings on the final set of items were all very high, and so extensive item pruning was not necessary. This pattern likely reflects the nature of the initial item pool: the items in FIBQ were either directly adapted from previously validated versions of the IBQ or were modified or generated based on other scales used in research on campus climate. Because the item pool was intentionally constructed to reflect other validated literature (rather than generated through broad exploratory item writing), the initial set was relatively focused and coherent.

Results of both studies yielded significant associations with other established and relevant psychological constructs, supporting the validation of the FIBQ. The FIBQ was significantly negatively correlated with institutional trust and with perceptions of procedural justice. These strong inverse relationships provide support for the concurrent validity of the FIBQ, suggesting that individuals who fear institutional betrayal are also more likely to perceive institutional authorities as procedurally unjust. Finally, the FIBQ was not meaningfully associated with just world beliefs or generalized anxiety, supporting the overall discriminant validity of the scale.

## Group-Based Differences in Fear of Institutional Betrayal

Group-based difference tests were used to evaluate known-groups validity and theoretical consistency (Davidson, 2014) of the construct of fear of institutional betrayal. Examining whether FIBQ scores differed across theoretically relevant groups acted as one form of evidence supporting the general construct of fear of institutional betrayal. These studies found preliminary evidence that fear of institutional betrayal is more salient in some marginalized groups, including sexual minorities, racial and ethnic minorities, and students with disabilities. Future research might explore potential causal mechanisms for higher fear of institutional betrayal in these groups, although these trends might reasonably be related to systemic barriers and historical injustices against these communities that undermine trust in these settings (Gómez, 2023; Kettrey et al., 2026; Nightingale, 2021; Rosenthal et al., 2016; Webermann & Holland, 2022).

This study aligns with research showing that students with diverse social identities may differ in how they perceive the adequacy of institutional responses to sexual violence (Dufour, 2024; Kettrey et al., 2026; Nightingale, 2021; Smidt et al., 2021; Smith et al., 2016). Future research should build on this: literature indicates that multiple marginalization (Cyrus, 2017) puts many individuals at higher risk of experiencing sexual violence during their lifetimes (Wilkins et al., 2023), and also exacerbates the disproportionate impact of sexual violence on survivors’ health and wellbeing (Cyrus, 2017; Kettrey et al., 2026; Howard Valdivia et al., 2023; Wilkins et al., 2023). Multiple marginalization is also associated with increased risk of receiving poor social and institutional responses to that violence (Gómez, 2023; Gómez et al., 2023; Hibberd, 2017; Howard Valdivia et al., 2023), and thus warrants exploration in future research on fear of institutional betrayal.

Next, in both studies, first-year students had significantly lower fear of institutional betrayal than students in upper years. In addition, there was no difference in fear of institutional betrayal scores for mature students (compared to non-mature students), first-generation students (compared to non-first-generation students), or students who had moved away from home to attend school (compared to those who did not). However, Study 2 also demonstrated a significant relationship between age and fear of institutional betrayal, with older students generally reporting lower fear of institutional betrayal. Similarly, international students were lower in fear of institutional betrayal than domestic students. Finally, Study 2 also found that college students had less fear of institutional betrayal than university students. One tentative explanation for this particular finding is that university students may be more aware of institutional failures in responding to sexual violence compared to college students, given the greater visibility of such cases in university settings and of sexual violence prevention education more generally. In contrast, smaller college environments may foster a stronger sense of support and community. Similarly, international students may hold different cultural expectations regarding institutional responsibility, which might contribute to less fear of institutional betrayal. These tentative interpretations require further investigation in future research.

Finally, victims of sexual violence overall (in Study 1) and victims of rape (in Study 2) reported higher perceptions of fear of institutional betrayal than non-victims. However, post-hoc tests in both studies revealed no differences based on different types of experiences (e.g., rape versus coercion). On a conceptual level, this pattern suggests that although fear of institutional betrayal is not reducible to victimization (given that non-victims also report these perceptions), the experience of victimization may exacerbate fear. Further research is necessary to conclusively identify systemic factors that predict fear of institutional betrayal. However, it is possible that victimization experience corresponds to greater direct or indirect exposure to institutional response systems, which may shape victims’ expectations about how such systems function. Women who are victims of sexual violence may receive any number of supportive or harmful responses to victimization disclosures (Ullman et al., 2017). For women who received negative responses to past disclosures, the experience may lead to increased fear of receiving negative responses again. Future research should explore perceptions of support in response to violence disclosures (e.g., The Social Reactions Questionnaire; Ullman et al., 2017) in the relation between victimization and fear of institutional betrayal.

Alternatively, women with sexual violence victimization experiences may know others who have had poor experiences, or may simply show increased attention to peer narratives about institutional response. A study by Hannan and MacDonald (2023) demonstrated that survivors of sexual violence were more likely to engage with social media platforms focused on survivors’ experiences, such as pages for survivors to share their stories. Survivors who viewed this type of content also reported higher institutional betrayal. The authors concluded that high exposure to this content may heighten perceptions of institutional betrayal (a pattern that would extend to fear of institutional betrayal). This also supports the idea that fear of institutional betrayal is deeply related to social norms and cultural discourse about sexual violence.

Nevertheless, non-victims also report fear of institutional betrayal, suggesting this is an attitude that can be held by anyone within a university community. It is an anticipatory or vicariously learned belief about institutional responses, shaped by perceived norms, peer stories, and broader campus culture. Ultimately, the FIBQ may complement (rather than replicate) sexual violence victimization measures and highlight perceptions of all members of an institutional community, regardless of victimization history.

Last, it is important to note that the measure of victimization in the current studies asked about lifetime experiences of victimization rather than exclusively recent or campus-specific experiences. This may have weakened observed associations with fear of institutional betrayal, as assaults occurring long ago or outside of the university context might be less likely to shape current institutional perceptions. Future research should test whether victimization during enrollment more consistently predicts fear of institutional betrayal.

## Strengths and Limitations

The current paper included two studies with different samples. The capacity to replicate and extend findings from Study 1 with a larger, more diverse, and national sample in Study 2 is a strength, especially given the inclusion of college students, which is rare in Canadian post-secondary research on sexual violence. The two sample sizes were also both sufficiently powered and diverse. However, these studies have different limitations. In Study 1, the sample was limited to students at one Ontario university, which may have led to low variability. Moreover, if the institutional climate around sexual violence was particularly good or bad, this culture would naturally affect campus-wide perceptions of fear of institutional betrayal. In a Canadian survey of university students, Maclean (2020) reported data on student satisfaction in a number of areas (e.g., residence life), with the University of Windsor ranking number 1 in students’ perceptions of sexual violence prevention efforts compared to other comprehensive (i.e., graduate and undergraduate) institutions in Canada. This suggests that, as recently as 2020, the climate around perceptions of sexual violence prevention at this particular institution was relatively positive. Although this is encouraging news for students at the institution, a generally positive or negative climate may affect psychometric assessments of the scale. However, this issue was mitigated by the sample used for Study 2, which was not specific to any single institution. However, crowdsourced panel data has its own drawbacks. Recent research by Novielli et al. (2025) examined differences in data quality between Qualtrics Panel samples and university student samples. Authors noted that Qualtrics Panel data tends to be of lower quality (e.g., higher rate of failure on attention checks, shorter completion time, and more evidence of inattentive responding). Indeed, the data obtained for the current study required a higher degree of cleaning than the student sample at University of Windsor, and many responses were excluded due to failed attention checks and low-quality responding. These concerns are not unique to crowdsourced panels. However, they underscore the importance of interpreting findings with some caution and of replicating results with samples collected through alternative recruitment strategies. It is also worth noting that although Study 2 included attention checks, Study 1 did not.

A final limitation of the current research is the exclusive focus on the experiences of women (and some gender-diverse participants in Study 1). This initial validation purposely focused on women in post-secondary settings, given the established prevalence rates of sexual violence and institutional betrayal for this population. Accordingly, the present findings should be interpreted as primarily applicable to women. Men may differ in their perceptions of institutional trust and in the extent to which they fear harm from their institution. Fear is contingent on perceived risk of victimization. As noted, men are typically less likely to assume sexual violence will happen to them at all (Chapin & Pierce, 2012; Lane et al., 2009). Theoretically, men may not experience fear of institutional betrayal in the same way as women, or may be less likely to believe that institutional betrayal could happen to them personally. Moreover, the ways men experience (fear of) institutional betrayal may, theoretically, differ from the ways women experience it. For example, men's concerns may center on false accusation, stigma, or disbelief of victimization, potentially representing distinct subscales of fear of institutional betrayal, and thus warranting distinct psychometric analysis. Such an analysis was out of scope for the present study, but it is a relevant direction for future research on fear of institutional betrayal.

## Practical Implications

The current studies raise several relevant considerations for future research. First, fear of institutional betrayal is a construct relevant to all post-secondary students and is not exclusive to survivors of violence. However, given the conceptual association with sexual violence, future research might seek to further explore perceptions of fear of institutional betrayal specifically in survivor populations, such as in conjunction with the IBQ.2 (Smith & Freyd, 2017) or IBSQ (Rosenthal et al., 2016). This would allow for a deeper examination of the extent to which fear of institutional betrayal aligns with lived institutional betrayal experiences. Next, in Study 1, fear of institutional betrayal was higher among minoritized students than non-minoritized peers. Given that this finding was not replicated in Study 2, future research is warranted to determine whether and why racialized students might perceive their institutions differently from non-racialized peers. Accordingly, future research should test the measurement invariance of the FIBQ across diverse groups to ensure that any observed group differences reflect true differences in fear of institutional betrayal, as opposed to differences in how the construct is measured. In addition, a limitation of the current studies is that racial minority status was assessed through a binary self-report question, which asked participants to indicate whether they identified as racialized. Therefore, future research using the FIBQ might consider a more nuanced approach to measuring ethnicity and racial identity.

The FIBQ represents a potentially important tool for feminist psychologists and gender scholars to quantify and examine campus climate around sexual violence and student perceptions of post-secondary institutions’ capacity to respond to these issues. In addition to academic research, one of the most promising future applications of this new tool is campus climate assessment, such as the Administrator-Researcher Campus Climate Collaborative (ARC3) survey (Swartout et al., 2019) or similar instruments. It could be valuable to post-secondary institutions that wish to better understand the extent to which campus community members trust that their institution is equipped to respond to sexual violence. The FIBQ may be particularly helpful in identifying specific student concerns regarding safety, fairness, and accountability in institutional responses to harm. Moreover, given that fear of institutional betrayal includes concerns about systemic neglect of sexual violence (e.g., not taking actions to address factors that contribute to sexual violence), the FIBQ may help institutions identify which target areas of institutional response require immediate attention. The current findings support the use of the FIBQ as a valuable tool in both future academic research and in applied work, such as program evaluation or climate assessments.

## Conclusion

Fear of institutional betrayal represents the belief that institutions will fail to respond supportively to instances of campus sexual violence, that engaging with institutional processes might result in harm to survivors, and that institutions are not effective at preventing sexual violence. In the present paper, two studies provided initial (but robust) evidence for the psychometric soundness of the FIBQ, a new tool for measuring this attitude. The initial validation study (focused on the perceptions of post-secondary women students) demonstrated that the scale is internally reliable and supports a unidimensional factor structure. Replication in a larger, more diverse, Canadian sample confirmed these findings and enabled the revision and refinement of items, resulting in reliable responses to this construct. Validity was supported through theoretically consistent correlations with related and unrelated constructs across both samples. Future work should explore fear of institutional betrayal in other samples, such as men, and aim to validate the tool with those samples as well. Nevertheless, these studies demonstrate that the FIBQ is a psychometrically sound instrument that captures a construct not previously assessed in literature.

## Supplemental Material

sj-docx-1-pwq-10.1177_03616843261449772 - Supplemental material for Development, Reliability, and Initial Validation of the Fear of Institutional Betrayal Questionnaire Supplemental material, sj-docx-1-pwq-10.1177_03616843261449772 for Development, Reliability, and Initial Validation of the Fear of Institutional Betrayal Questionnaire by Gena K. Dufour in Psychology of Women Quarterly
