# Supplementary material for: Development, Reliability, and Initial Validation of the Fear of Institutional Betrayal Questionnaire
Source: Psychol Women Q. 2026 May 13;50(3):320–42. doi: 10.1177/03616843261449772 (PMC13436808; doi:10.1177/03616843261449772)
Supplement: Supplementary file 1 [file sj-docx-1-pwq-10.1177_03616843261449772.docx]

**Supplemental Materials: Fear of Institutional Betrayal Questionnaire (FIBQ)**

Please indicate your level of agreement to the following statements using the following rating scale:

1 = Strongly Disagree

2 = Disagree

3 = Somewhat Disagree

4 = Neither Agree nor Disagree

5 = Somewhat Agree

6 = Agree

7 = Strongly Agree

| Fear of Institutional Betrayal Questionnaire (FIBQ) | | | |
| --- | --- | --- | --- |
| **Item number** | **Item** | **Item modified from original source(s)** | **Other supporting evidence** |
| 1 | I worry that, in my university, people who report sexual violence are sometimes punished or experience other negative consequences. | Foster & Fullagar, 2018; Lind et al., 2020; McMahon et al., 2015; Smith & Freyd, 2013; White House Task Force to Protect Students from Sexual Assault, 2014 | Ahern, 2018; Boyle et al., 2017; Spencer et al., 2017 |
| 2 | I believe my university doesn’t take reports of sexual violence seriously. | Herres et al., 2021; Holland, 2019; McMahon et al., 2015; White House Task Force to Protect Students from Sexual Assault, 2014 | Tredinnick, 2022; Rosenthal et al., 2017 |
| 3 | I worry my university creates an environment in which sexual violence seems common or normal. | Lind et al., 2020; Smith & Freyd, 2013 | Krausch, 2019; Riley, 2019 |
| 4 | I’m afraid that victims/survivors of sexual violence aren’t treated with dignity and respect by my university. | Holland, 2020 | Amar et al., 2014; Brink et al., 2021; Webermann & Holland, 2022 |
| 5 | I worry that my university does not care about sexual violence. | Annelise et al., 2020; Gartner et al., 2023; Webermann et al., 2023 | Brink et al., 2021 |
| 6 | I’m afraid that when sexual violence is reported, my university is only concerned with protecting its reputation. | Lind et al., 2020; Smith & Freyd, 2017 | Nightingale & Cousineau, 2024; Works et al., 2021 |
| 7 | I believe that when sexual violence is reported, my university sometimes mishandles cases. | Lind et al., 2020; Smith & Freyd, 2017 | Rosenthal et al., 2017; Tredinnick, 2022 |
| 8 | I believe that university administrators deny that sexual violence occurs on campus. | Lind et al., 2020; Smith & Freyd, 2017 | Riley, 2019; Works et al., 2021 |
| 9 | I worry that my university wouldn’t punish someone who engaged in sexual violence on campus. | White House Task Force to Protect Students from Sexual Assault, 2014 | Bedera, 2023; Mascagni, 2017 |
| 10 | I’m afraid that people who report sexual violence in my institution won’t be believed. | Foster & Fullagar, 2018 | Boyle et al., 2017; Spencer et al., 2017 |
| 11 | My university creates an environment in which sexual violence seems more likely to occur. | Lind et al., 2020; Smith & Freyd, 2013 | Crocker et al., 2020 |
| 12 | I worry that my university isn’t taking steps to prevent sexual violence. | Lind et al., 2020; Smith & Freyd, 2013 | Bonar et al., 2022; Cherniawsky & Morrison, 2022 |
| 13 | I’m afraid that reporting sexual violence might lead to retaliation by my university. | Foster & Fullagar, 2018; McMahon et al., 2015; White House Task Force to Protect Students from Sexual Assault, 2014. | Ahern, 2018; Boyle et al., 2017 |
| 14 | I worry that the university is too slow to respond to incidents of sexual violence on campus. | Rizzo et al., 2021; Sulkowski, 2011 | Webermann et al., 2023 |
| 15 | I worry that my university wouldn’t maintain the privacy of people who report sexual violence. | Herres et al., 2021; Holland, 2019; McMahon et al., 2015; White House Task Force to Protect Students from Sexual Assault, 2014 | Amar et al., 2014; Atreyi et al., 2022; Hibberd, 2017; Javorka & Campbell, 2019 |
| 16 | I believe that my university sometimes works to cover up instances of sexual violence on campus. | Lind et al., 2020; Smith & Freyd, 2013 | Webermann et al., 2023; Works et al., 2021 |
| 17 | I am afraid that people who report sexual violence on campus are labeled as troublemakers. | McMahon et al., 2015; White House Task Force to Protect Students from Sexual Assault, 2014 | Khan et al., 2018; Lorenz et al., 2022 |
| 18 | I worry that victims/survivors who report sexual violence in my university will be blamed. |  | Smith & Freyd, 2014; Sall & Littleton, 2022; Spencer et al., 2017; Stader & Williams-Cunningham, 2017 |
| 19 | I worry that my university doesn’t take action to address specific factors that may lead to sexual violence. | McMahon et al., 2015; White House Task Force to Protect Students from Sexual Assault, 2014 | Brink et al., 2021 |
| 20 | I worry that my university would make it difficult or complicated to report sexual violence. | Lind et al., 2020; Smith & Freyd, 2013 | Brubaker, 2009 |
| 21 | I fear that some people who report sexual violence would receive a poor response from the university because of their identity (e.g., race, ethnicity, sexuality, or gender-identity, or pronouns). |  | Abdmolaei, 2020; Hibberd, 2017; Karunaratne, 2021; Nightingale, 2022 |
| 22 | I don’t believe my university provides enough support to victims/survivors of sexual violence. | McMahon et al., 2015; White House Task Force to Protect Students from Sexual Assault, 2014 | Stewart, 2021 |
| 23 | I worry that university representatives (e.g., staff, administrators) probably wouldn’t be able to do anything to help victims/survivors of sexual violence. |  | Brink et al., 2021; Hibberd, 2017; Nightingale, 2021 |

**References for the FIBQ**

Abdmolaei, S. (2020). *Racialized women’s experiences of sexual violence and harassment in Canadian higher education: An intersectional analysis*. (Doctoral dissertation from The University of Western Ontario). ProQuest Dissertations and Theses Global.

Ahern, K. (2018). Institutional betrayal and gaslighting: Why whistle-blowers are so traumatized. *Journal of Perinatal and Neonatal Nursing*, *32*(1), 59–65. <https://doi.org/10.1097/JPN.0000000000000306>

Amar, A. F., Strout, T. D., Simpson, S., Cardiello, M., & Beckford, S. (2014). Administrators’ perceptions of college campus protocols, response, and student prevention efforts for sexual assault. *Violence and Victims*, *29*(4), 579–593. <https://doi.org/10.1891/0886-6708.VV-D-12-00154>

Annelise, M., Geiger, E., & Brewster, M. (2020). Interpersonal Violence Prevention Considerations for Sexual Minority College Students: Lower Campus Connection, Worse Perceptions of Institutional Support, and more Accurate Understandings of Sexual Consent. *Journal of Family Violence*, *35*(6), 589–601. <https://doi.org/https://doi.org/10.1007/s10896-019-00089-5>

Atreyi, M., Dallas, S., Stephanie, S., Raine, S. C., Bloom, B. E., & Wagman, J. A. (2022). Structural barriers to accessing the Campus Assault Resources and Education (CARE) offices at the University of California (UC) campuses. *Journal of Interpersonal Violence*, *37*(21–22). <https://doi.org/10.1177/08862605211042813>

Bedera, N. (2023). I can protect his future, but she can’t be helped: Himpathy and hysteria in administrator rationalizations of institutional betrayal. *Journal of Higher Education*, *(advance online),* 1–24. <https://doi.org/10.1080/00221546.2023.2195771>

Bonar, E. E., DeGue, S., Abbey, A., Coker, A. L., Lindquist, C. H., McCauley, H. L., Miller, E., Senn, C. Y., Thompson, M. P., Ngo, Q. M., Cunningham, R. M., & Walton, M. A. (2022). Prevention of sexual violence among college students: Current challenges and future directions. *Journal of American College Health*, *70*(2), 575–588. <https://doi.org/10.1080/07448481.2020.1757681>

Boyle, K. M., Barr, A., & Clay-Warner, J. (2017). The effects of feminist mobilization and women’s status on universities’ reporting of rape. *Journal of School Violence*, *16*(3), 317–330. <https://doi.org/10.1080/15388220.2017.1318580>

Brink, J. G., Mdaka, Q., Matee, L., & Weppelman, K. (2021). Practitioner’s perspectives on a national South African higher education institution policy framework mitigating gender-based violence at a South African university: Governance, implementation challenges and opportunities. *International Journal of Critical Diversity Studies*, *4*(2), 47–60. <http://doi.org/10.0.51.113/intecritdivestud.4.2.0047>

Brubaker, S. J. (2009). Sexual assault prevalence, reporting and policies: Comparing college and university campuses and military service academies. *Security Journal*, *22*(1), 56–72. <https://doi.org/10.1057/sj.2008.10>

Cherniawsky, S., & Morrison, M. (2022). “You Should Have Known Better”: The Social Ramifications of Victimization-Focused Sexual Assault Prevention Tips. *Journal of Interpersonal Violence*, *37*(1–2), NP125–NP146. <https://doi.org/10.1177/0886260520913650>

Crocker, D., Minaker, J., & Neland, Amanda. (2020). *Violence Interrupted: Confronting Sexual Violence on University Campuses*. McGill-Queen’s Press-MQUP.

Foster, P. J., & Fullagar, C. J. (2018). Why don’t we report sexual harassment? An application of the theory of planned behavior. *Basic and Applied Social Psychology*, *40*(3), 148–160. <https://doi.org/10.1080/01973533.2018.1449747>

Gartner, R. E., Ballard, A. J., Smith, E. K., Risser, L. R., Chugani, C. D., & Miller, E. (2023). “There’s no safety in these systems”: Centering trans and gender diverse students’ campus climate experiences to prevent sexual violence. *Journal of Diversity in Higher Education*. <https://doi.org/10.1037/dhe0000512>

Herres, J., Wang, S. B., Bobchin, K., & Draper, J. (2021). A socioecological model of risk associated with campus sexual assault in a representative sample of liberal arts college students. *Journal of Interpersonal Violence*, Vol. 36. <https://doi.org/https://doi.org/10.1177/0886260518785376>

Hibberd, A. J. D. (2017). *How university policymakers problematize sexual violence on their campus: A policy discourse analysis.* (Masters thesis from McGill University). ProQuest Dissertations and Theses Global.

Holland, K. J. (2019). Examining responsible employees’ perceptions of sexual assault reporting requirements under federal and institutional policy. *Analyses of Social Issues and Public Policy (ASAP)*, *19*(1), 133–149. <https://doi.org/10.1111/asap.12176>

Holland, K. J. (2020). Correlates of College Women’s Intentions to Use Formal Campus Supports for Sexual Assault. *Psychology of Violence*, *10*(2), 245–254. <https://doi.org/10.1037/vio0000240>

Javorka, M., & Campbell, R. (2019). Advocacy services for college victims of sexual assault: navigating complicated confidentiality concerns. *Journal of Trauma and Dissociation*, *20*(3), 304–323. <https://doi.org/10.1080/15299732.2019.1571889>

Karunaratne, N. (2021). Fostering (re)connections: South Asian students healing from dating violence. *Journal of Diversity in Higher Education*, *16*(2), 131–143. <https://doi.org/10.1037/dhe0000331>

Khan, S. R., Hirsch, J. S., Wamboldt, A., & Mellins, C. A. (2018). “I didn’t want to be 'that girl”’: The social risks of labeling, telling, and reporting sexual assault. *Sociological Science*, *5*(1), 432–460. <https://doi.org/10.15195/v5.a19>

Krausch, M. (2019). “Every Breath You Take”: The university crisis as normalized feminist exclusion. *Feminist Formations, 31*(1), 20–44. <https://muse.jhu.edu/article/723566>

Lind, M. N., Adams-Clark, A. A., & Freyd, J. J. (2020). Isn’t high school bad enough already? Rates of gender harassment and institutional betrayal in high school and their association with trauma-related symptoms. *PLOS ONE*, *15*(8). <https://doi.org/10.1371/journal.pone.0237713>

Lorenz, K., Hayes, R., & Jacobsen, C. (2022). “Keeping the Wound Open”: Survivor Experiences with Title IX Investigations. *Women and Criminal Justice*, *0*(0), 1–21. <https://doi.org/10.1080/08974454.2022.2060896>

Mascagni, B. (2017). Rape, apology, and the business of title IX compliance. *Politics, Groups and Identities*, *5*(1), 182–196. <https://doi.org/10.1080/21565503.2016.1273123>

McMahon, S., Stepleton, K., O’Connor, J., & Cusano, J. (2015). iSPEAK student experiences, attitudes, and beliefs about sexual violence: Results of the Rutgers University-New Brunswick campus climate assessment. <http://endsexualviolence.rutgers.edu/wp-content/uploads/pdf/2018_iSPEAK_NB_Campus-Climate_Findings_Full_Report.pdf>

Nightingale, S. (2021). “It probably hurt more than it helped”: LGBTQ survivors of sexual assault and their experience with the college Title IX reporting process. *Advances in Social Work*, *21*(4), 1280–1299. <https://doi.org/10.18060/25211>

Nightingale, S. D. (2022). The role of trust in perceptions of the sexual assault reporting climate for LGBQ college students. *Journal of Diversity in Higher Education*, *15*(6), 790–800. <https://doi.org/10.1037/dhe0000317>

Nightingale, S. D., & Cousineau, S. (2024). Institutional Courage in the college context: A mixed-methods analysis of campus victim advocate perceptions and experiences. *Violence Against Women*. <https://doi.org/10.1177/10778012241234893>

Riley, D. (2019). Pipelines, Persistence, and Perfidy: Institutional Unknowing and Betrayal Trauma in Engineering. *Feminist Formations*, *31*(1), 1–19. <https://doi.org/10.1353/ff.2019.0006>

Rizzo, A. J., Demers, J. M., Howard, M. E., & Banyard, V. L. (2021). Perceptions of campus authorities: Institutional responses, fairness, and bystander action. *Journal of American College Health*, *69*(8), 851–859. <https://doi.org/10.1080/07448481.2020.1711762>

Rosenthal, M., Smith, C. P., & Freyd, J. J. (2017). Behind closed doors: University employees as stakeholders in campus sexual violence. *Journal of Aggression, Conflict and Peace Research*, *9*(4), 290–304. <https://doi.org/https://doi.org/10.1108/JACPR-02-2017-0272>

Sall, K., & Littleton, H. (2022). Institutional Betrayal: A mixed methods study of college women’s experiences with on-campus help-seeking following rape. *Journal of Trauma and Dissociation*, *23*(5), 584–601. <https://doi.org/10.1080/15299732.2022.2079795>

Smith, C. P., & Freyd, J. (2013). Dangerous safe havens: Institutional betrayal exacerbates sexual trauma. *Journal of Traumatic Stress*, *20*(3), 251–262. <https://doi.org/10.1002/jts>

Smith, C. P., & Freyd, J. J. (2014). Institutional betrayal. *American Psychologist*, *69*(6), 575–584. <https://doi.org/10.1037/a0037564>

Smith, C. P., & Freyd, J. J. (2017). Insult, then injury: Interpersonal and institutional betrayal linked to health and Dissociation. *Journal of Aggression, Maltreatment and Trauma*, *26*(10), 1117–1131. <https://doi.org/10.1080/10926771.2017.1322654>

Spencer, C., Mallory, A., Toews, M., Stith, S., & Wood, L. (2017). Why sexual assault survivors do not report to universities: A feminist analysis. *Family Relations*, *66*(1), 166–179. <https://doi.org/https://doi.org/10.1111/fare.12241>

Spencer, C., Mallory, A., Toews, M., Stith, S., & Wood, L. (2017). Why Sexual Assault Survivors Do Not Report to Universities: A Feminist Analysis. *Family Relations*, *66*(1), 166–179. <https://doi.org/https://doi.org/10.1111/fare.12241>

Stader, D. L., & Williams-Cunningham, J. L. (2017). Campus Sexual Assault, Institutional Betrayal, and Title IX. *Clearing House: A Journal of Educational Strategies, Issues and Ideas*, *90*(5–6), 198–202. <https://doi.org/10.1080/00098655.2017.1361287>

Stewart, T. J. (2021). “Dear higher education, there are sex workers on your campus”: Rendering visible the realities of U.S. college students engaged in sex work. *Journal of Diversity in Higher Education*. <https://doi.org/10.1037/dhe0000351>

Sulkowski, M. L. (2011). An investigation of students’ willingness to report threats of violence in campus communities. *Psychology of Violence*, *1*(1), 53–65. <https://doi.org/10.1037/a0021592>

Tredinnick, L. (2022). Sexual assault prevention with student-athletes: Exploring perceptions of the campus climate and awareness of sexual assault policies and resources. *Journal of Interpersonal Violence*, *37*(9–10). <https://doi.org/10.1177/0886260520967144>

Webermann, A. R., & Holland, K. J. (2022). Inconsistency is the consistency: The Title IX reporting process for sexual and gender-based misconduct within Maryland public universities. *Psychology of Women Quarterly*, *46*(4), 468–483. <https://doi.org/k3tc>

Webermann, A. R., Holland, K. J., & Murphy, C. M. (2023). Student experiences reporting sexual and gender-based misconduct to the Title IX Office at a public state university. *Violence Against Women*. <https://doi.org/10.1177/10778012221150274>

White House Task Force to Protect Students from Sexual Assault (2014). Climate Surveys: Useful Tools to Help Colleges and Universities in Their Efforts to Reduce and Prevent Sexual Assault. <https://drulibrary.uoregon.edu/sites/drulibrary2.uoregon.edu/files/rpds/entry/file_for_download/Climate_Surveys_-_Not_Alone.pdf>

Works, W. B. L. L., Wong, J. S., & Lee, C. (2021). Damage control: Canadian University crisis management responses to sexual assault. *Atlantic Journal of Communication*, *29*(1), 39–52. <https://doi.org/10.1080/15456870.2019.1698580>
